# Ultrasound-responsive Janus patch with mechanical anisotropy, pro-healing, and anti-adhesion properties for abdominal wall defect repair

**DOI:** 10.1186/s12951-025-03779-z

**Published:** 2025-11-05

**Authors:** Binying Peng, Binghua Ma, Hao Lu, Zixin Chen, Wenxuan Xiong, Hui Wang, Zhaopeng Cai, Xingxing Shi, Rongkang Huang

**Affiliations:** 1 Department of General Surgery (Colorectal Surgery), Guangdong Institute of Gastroenterology, Biomedical Innovation Center, Key Laboratory of Human Microbiome and Chronic Diseases (Sun Yat-sen University), Ministry of Education, Guangdong Provincial Key Laboratory of Colorectal and Pelvic Floor Diseases, The Sixth Affiliated Hospital, Sun Yat-sen University, Guangzhou, 510655 P. R. China; 2 The Eighth Affiliated Hospital, Sun Yat-sen University, Shenzhen, 518033 P. R. China; 3 Translational Medicine Research Center, Naval Medical University, Shanghai, 200433 P. R. China; 4 Department of Colorectal Surgery, Second Affiliated Hospital of Navy Medical University, Shanghai, 200003 P. R. China

**Keywords:** Patch, Anisotropy structure, Janus structure, Pro-healing, Anti-adhesion

## Abstract

Abdominal wall exhibits a complex anisotropic architecture with region-specific mechanical gradients, essential for maintaining biomechanical integrity during physiological deformations. However, most clinical patches possess isotropic structures and fail to meet the mechanical anisotropy requirements of abdominal wall. Herein, inspired by the hierarchical structure of abdominal wall, an innovative ultrasound-responsive Janus patch (JPLCL/ZnO) is designed by constructing a poly (2-methacryloyloxyethyl phosphorylcholine) (PMPC) coating on the top surface of a fibrous anisotropic patch (PLCL/ZnO) via multi-channel electrospinning and in situ photocuring technologies. By adjusting fiber orientation, our patch achieves tunable anisotropy ratios (1–14), matching the mechanical gradients of abdominal wall (1–9). The PMPC-coated side prevents tissue adhesion, while the anisotropic PLCL/ZnO side promotes directional cell growth and tissue regeneration under ultrasound stimulation. Moving toward clinical translation, we developed a large mammal defect model in pigs with abdominal wall resection. By integrating excellent mechanical anisotropy, pro-healing, anti-adhesion, and immune regulation properties, our JPLCL/ZnO patch demonstrates a significant advancement for abdominal wall defect repair.

**Graphical Abstract:**

**Figure:**
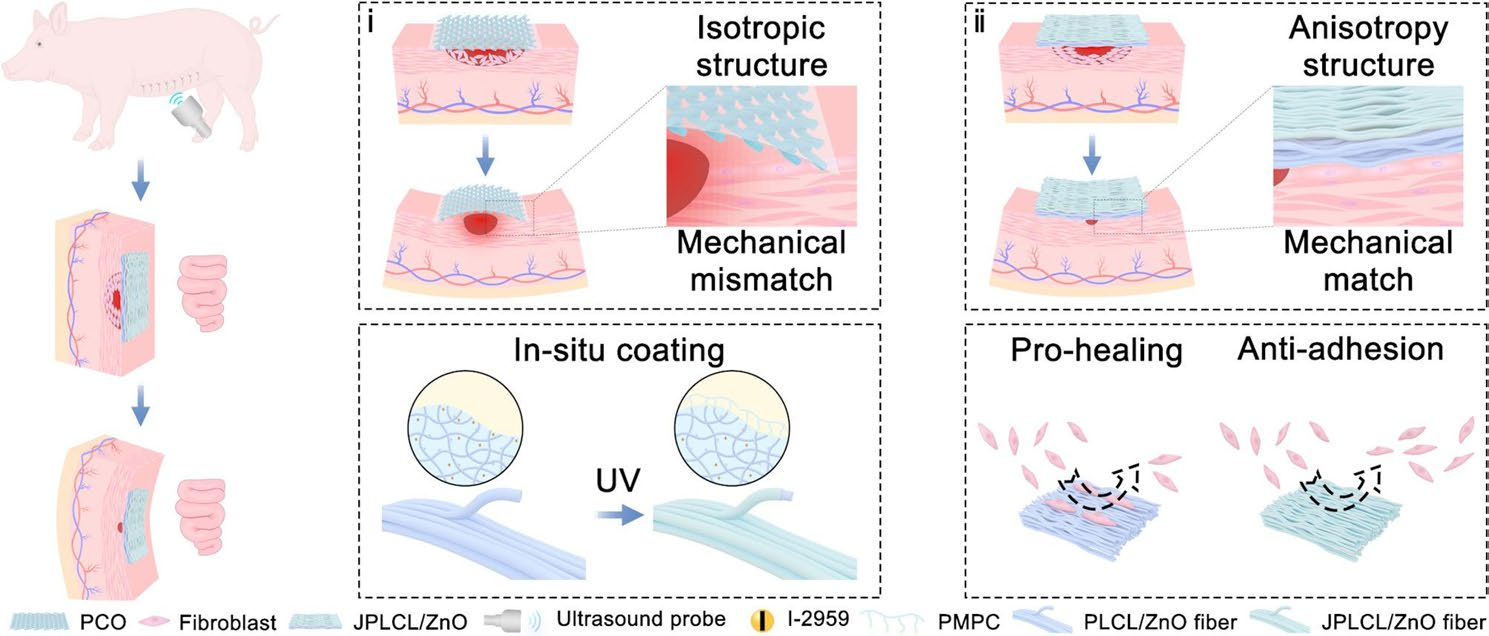
Graphical Abstract

**Supplementary Information:**

The online version contains supplementary material available at 10.1186/s12951-025-03779-z.

## Introduction

**Fig. 1:**
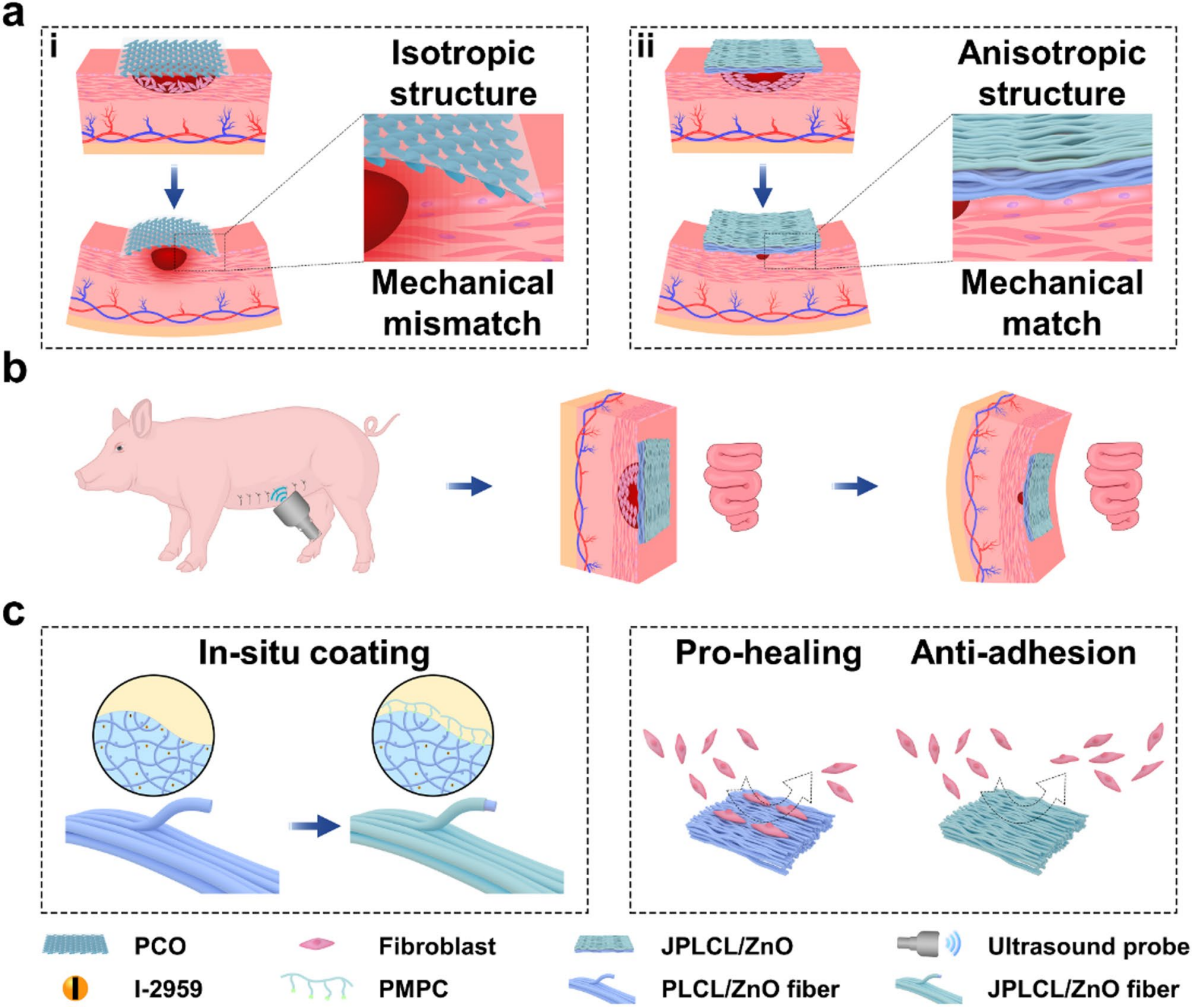
Schematic diagram of JPLCL/ZnO patch for abdominal wall defect repair with ultrasound therapy. **a**) Schematic diagram illustrating the biomechanical compatibility of clinical PCO patch and our JPLCL/ZnO patch with natural abdominal wall tissue: (**i**) PCO patch possesses an isotropic structure, which may lead to a mechanical mismatch with the anisotropic nature of abdominal wall tissue, potentially hindering tissue regeneration and increasing the risk of recurrence. (**ii**) Our JPLCL/ZnO patch possesses an anisotropic structure, which achieves a mechanical match with abdominal wall tissue, thereby facilitating tissue integration and promoting abdominal wall defect repair. **b**, **c**) By in-situ coating PMPC on each fiber of the top surface, our JPLCL/ZnO patch can promote abdominal wall defect repair in porcine model by integrating excellent mechanical anisotropy, pro-healing, and anti-adhesion properties

Abdominal wall is characterized by a complex anisotropic architecture consisting of orientation-dependent multilayered muscles and connective tissues [1–5]. This inherent tissue organization generates region-specific mechanical gradients [6, 7], with measured anisotropy ratios ranging from 1 to 3 in the lateral region [8], 4 to 6 in the rectus sheath [9], and 8 to 9 in the linea alba [10]. This structural hierarchy enables biomechanical integrity during physiological deformations associated with respiration and visceral pressure fluctuations [11]. Disruption of this continuity can lead to hernia, necessitating repair strategies that restore both tissue continuity and mechanical function of the abdominal wall. In clinical practice, the isotropic Parietex composite (PCO) patch is commonly used but often fails to match the anisotropic requirements of abdominal wall, potentially hindering functional tissue regeneration and increasing the risk of recurrence (Fig. 1) [12, 13]. Therefore, it is crucial to design biomimetic anisotropic patches that recapitulate region-specific mechanical gradients in abdominal wall defect repair.

Generally, the development of anisotropic patch faces dual challenges of biomechanical compatibility and biological functional adaptability. On one hand, various approaches have been explored to fabricate anisotropic patches, such as compression and stretching forces [14–18], directional crystallization [19–21], ion diffusion [22, 23], and photolithography [24, 25] for anisotropic hydrogel patches, as well as knitting and braiding techniques for fabric-based patches [26–28]. However, the anisotropy ratios of most patches are limited to a range of 1–3 [29, 30], which can not meet the full spectrum of abdominal wall anisotropy requirements (a range of 1–9) [8–10], thereby affecting tissue integration [31]. On the other hand, commercial PCO patch has successfully achieved asymmetric functions of promoting cell proliferation and preventing tissue adhesion by the construction of a Janus structure comprising a polyester and collagen bilayer. However, this bilayer design suffers from weak interfacial bonding and mechanical mismatch, which may lead to delamination, displacement, and poor tissue integration. In our previous work, we have successfully developed a series of porous polymer-based materials [31–33] that integrates pro-healing property and visceral anti-adhesion capability, but facing challenges such as isotropic limitations and poor biodegradability. Few studies have attempted to develop anisotropic Janus biodegradable patches that integrate biomechanical compatibility with multifunctional capabilities, but most of these studies have predominantly focused on small animal models, which are difficult to match biomechanical characterization of human body [31, 32, 34]. Therefore, it is still highly challenging to prepare an anisotropic patch that integrates biomechanical compatibility with regenerative biological functions, alongside with comprehensive validation in large animal experiments.

Herein, bioinspired by the abdominal wall anisotropic architecture, we have successfully developed an innovative ultrasound-responsive anisotropic patch through multi-channel electrospinning and in situ photocuring technologies, which integrates excellent mechanical anisotropy, pro-healing, anti-adhesion, and immune regulation properties to promote abdominal wall defect repair (Fig. 1). Specifically, by multi-channel electrospinning a polymer mixture containing poly (lactide-co-caprolactone) (PLCL), zinc oxide (ZnO) nanoparticles, and 2-hydroxy-4’-(2-hydroxyethoxy)-2-methylpropiophenone (I-2959) photoinitiator, a fibrous anisotropic patch (PLCL/ZnO) embedded with the photoinitiator is obtained. By immersing PLCL/ZnO patch in 2-methacryloyloxyethyl phosphorylcholine solution and conducting ultraviolet (UV) curing, a superlubricated poly (2-methacryloyloxyethyl phosphorylcholine) (PMPC) coating is uniformly grown on each fiber of the top surface, resulting in the formation of an ultrasound-responsive Janus patch (JPLCL/ZnO). ZnO nanoparticles are incorporated as piezoelectric nanofillers to enhance the piezoelectric response of PLCL, which enables the conversion of ultrasound-induced mechanical deformation into localized electrical signals and subsequently promotes tissue repair. Benefitting from its super lubrication characteristics, the PMPC-coated side facing the peritoneal cavity can effectively prevent tissue adhesion, while the anisotropic PLCL/ZnO side facing the abdominal wall can promote directional cell migration and proliferation to accelerate defect repair due to the anisotropic structure and the efficient guiding effect of electric fields under ultrasound therapy. Furthermore, by modifying the orientation stacking ratios of the fibers, our JPLCL/ZnO patch can achieve adjustable anisotropy ratios (1–14) to match the biomechanical properties of the abdominal wall tissue (1–9). Rat and porcine abdominal wall defect models further confirm that our JPLCL/ZnO patch can promote tissue repair in terms of mechanical match, tissue remodeling, anti-adhesion, and immune regulation properties. Therefore, our patch offers an attractive approach for designing biomimetic anisotropic materials for dynamic soft tissue repair.

## Materials and methods

### Materials

Poly (lactide-co-caprolactone) (PLCL, 50:50 mol%) was purchased from Jinan Daigang Biomaterial Co., Ltd. (China). Zinc oxide (ZnO, < 50 nm), benzophenone, erioglaucine disodium salt (85%), Triton X-100 solution (0.5% w/v), and 1,1,1,3,3,3-hexafluoro-2-propanol (HFIP) were purchased from Shanghai Macklin Biochemical Technology Co., Ltd. (China). 2-Methacryloyloxyethyl phosphorylcholine (MPC) was purchased from Shanghai Bide Pharmatech Co., Ltd. (China). 2-Hydroxy-4’-(2-hydroxyethoxy)-2-methylpropiophenone (I-2959) was purchased from Beijing G-CLONE Bio-Technology Co., Ltd. (China). Phosphate buffer saline (PBS) was purchased from Wuhan Servicebio Technology Co., Ltd. (China). Fetal bovine serum (FBS), high glucose dulbecco’s modified eagle medium (DMEM), and penicillin-streptomycin (PS) were purchased from Gibco-BRL Life Technologies, Inc. (USA). Cell counting kit-8 (CCK-8) and calcein AM/PI double staining kit were purchased from Beijing Solarbio Science & Technology Co., Ltd. (China). Hoechst 33342 staining solution and FITC phalloidin were purchased from Shanghai Beyotime Biotech Co., Ltd. (China). Lipopolysaccharide (LPS) was purchased from Biosharp (China). *Staphylococcus aureus* (*S. aureus*, ATCC 25923) and *Escherichia coli* (*E. coli*, ATCC 25922) were obtained from the department of laboratory medicine, Guangdong Provincial People’s Hospital (China). Standard fibroblast cell line L929 fibroblasts were provided from the Cell Bank of the Chinese Academy of Sciences (China). The male Sprague-Dawley rats and female Landrace pigs were provided from the Animal Center of Sun Yat-sen University (China) and Suzhou Zhenhu Medical Technology Co., Ltd. (China), respectively.

### Fabrication of JPLCL and JPLCL/ZnO patches

JPLCL and JPLCL/ZnO patches were fabricated by constructing a poly (2-methacryloyloxyethyl phosphorylcholine) (PMPC) coating on the top surface of fibrous anisotropic PLCL and PLCL/ZnO patches, respectively, via multi-channel electrospinning and in situ photocuring technologies. In this design, PLCL matrix serves as a flexible and biocompatible scaffold with intrinsic piezoelectricity that converts ultrasound-induced mechanical deformation into localized electrical signals, while ZnO nanoparticles are incorporated as piezoelectric nanofillers to further enhance the piezoelectric response.

Briefly, 8 g of PLCL and 0.08 g of I-2959 were added into 80 mL of HFIP solvent and completely dissolved to form a uniform PLCL solution. ZnO nanoparticles at different contents (0, 1, 3, and 5 wt%) was then added to PLCL solution and stirred overnight under 500 r min^−1^ in the dark. The mixture was evenly transferred into two syringes and then injected into two sets of 8-needle electrospinning spinnerets for electrospinning. The electrospinning process was carried out with an injection rate of 5 mL h^−1^ and a voltage of 16 kV. The fibrous PLCL or PLCL/ZnO film was collected using aluminum foil at a drum rotation speed of 1000 rpm and dried at 25 °C in a vacuum oven to remove the organic solvents.

The as-fabricated PLCL or PLCL/ZnO film (10 cm × 10 cm) was placed at the bottom of square petri dish, with a hollow square glass frame used to press the edges of the film. A 10% MPC solution was prepared by dissolving 5 g of MPC in 50 mL of deionized water, which was then added to the petri dishes to fully immerse the films. After photocuring with a UV-curing lamp (48 W) for 20 min, the films were thoroughly rinsed with sufficient deionized water at least 5 times to remove unreacted monomers. The films were then covered with a piece of quartz glass and dried at 25 °C in a vacuum oven. Finally, JPLCL and JPLCL/ZnO patches were obtained.

### Fabrication of JPLCL/ZnO patch with different orientation stacking ratios

8 g of PLCL, 0.08 g of I-2959, and 0.4 g (i.e., 5 wt%) of ZnO nanoparticles were added into 80 mL of HFIP solvent to form the polymer mixture. A portion of the mixture was electrospun and collected using aluminum foil at a drum rotation speed of 1000 rpm to obtain a film labeled as the 0° direction. The aluminum foil was then rotated by 90°, and the remaining mixture was continuously electrospun to obtain a second film labeled as the 90° direction. The volumes of the polymer mixture electrospun in the 0° and 90° directions (Table S1) were adjusted to prepare PLCL/ZnO films with different orientation stacking ratios (0°:90° = 10:1, 5:1, 3:1, and 1:1). After electrospinning, the as-fabricated PLCL/ZnO films were subsequently dried at 25 °C in a vacuum oven to remove the organic solvents. Finally, the dried films were immersed in a 10% MPC solution and photocured under a UV-curing lamp (48 W) for 20 min to obtain JPLCL/ZnO patches with different orientation stacking ratios.

### Fabrication of BPLCL/ZnO patch

8 g of PLCL and 0.4 g of ZnO nanoparticles were added into 80 mL of HFIP solvent and stirred overnight under 500 r min^−1^. The resulting mixture was loaded into two syringes and electrospun through two sets of 8-needle electrospinning spinnerets at an injection rate of 5 mL h^−1^ under a voltage of 16 kV. The film was collected at a drum rotation speed of 1000 rpm and dried at 25 °C in a vacuum oven. The dried films were then placed at the bottom of square petri dish, with a hollow square glass frame used to press the edges. A 10% benzophenone solution was prepared by dissolving 5 g of benzophenone in 50 mL of ethanol, which was added to the petri dish to fully immerse the films. After photocuring with a UV-curing lamp (48 W) for 20 min, the films were thoroughly rinsed with sufficient deionized water at least 5 times to remove unreacted monomers, then covered with a piece of quartz glass and dried at 25 °C in a vacuum oven. Finally, BPLCL/ZnO patch was obtained.

### Morphology characterization

The morphology and elemental mapping image were investigated by a field emission scanning electron microscope (SEM, Hitachi S-4800, Japan) at an acceleration voltage of 5 kV. The average fiber diameter and fiber orientation distribution in the SEM image were analyzed using Image J, where the maximum diameter of 50 fibers was randomly measured.

### Water contact angle characterization

Water contact angles were measured on a drop shape analyzer (Kruss DSA100, Germany).

### X-ray photoelectron spectroscopy characterization

X-ray photoelectron spectroscopy (XPS, ThermoFisher-VG Scientific ESCALAB 250Xi, USA) with a standard Al Ka X-ray source was used to analyze the chemical structure.

### Tensile testing

Tensile tests were conducted on a universal mechanical testing machine (WD-5A, Guangzhou Experimental Instrument Factory, China) at a stretching speed of 5 mm min^−1^. All samples were cut into rectangular strips (20 mm × 10 mm), and the loading direction was parallel or perpendicular to the fiber orientation of the samples.

### Electrical performance characterization

The electrical performances of JPLCL and JPLCL/ZnO patches (2 cm × 2 cm) were evaluated using a Mettler Sonicator (ME740, USA) at a power intensity of 0.5 W cm^−2^ and a pulse duty cycle of 50%. The patches were sandwiched between two copper electrodes and sealed with Ecoflex to protect against mechanical and humidity damage. The output voltage under ultrasound stimulation was recorded using a Rigol DS1102E oscilloscope. For further therapeutic evaluation of JPLCL/ZnO patch for defect repair in animal models, electrodes were removed before in vivo application.

### In vitro biocompatibility

The cytocompatibility was assessed by CCK-8 and live/dead staining assays. JPLCL/ZnO patches were sterilized under ultraviolet light for 1 h and then immersed in a culture medium (1 mL cell culture medium for 6 cm^2^ sample) at 37 ℃ for 24 h and filtered through a 0.4 μm filter to obtain sample extract solutions. L929 fibroblasts were seeded at a density of 3.0 × 10^3^ cells per well with 100 µL of DMEM medium containing 10% FBS and 1% PS. After 24 h of incubation, L929 fibroblasts adhered to the plates and the culture medium was then replaced with 100 µL of fresh culture medium as the control group or 100 µL of sample extracts as the experimental group. For CCK-8 assay, cells were washed three times with PBS solution and then 100 µL of DMEM with 10% CCK-8 was added into each well. After incubation for 1 h in an incubator with 5% CO_2_ at 37 °C, the optical density at a wavelength of 450 nm was measured with a microplate reader (VarisoskanLUX, Thermo Fisher) to determine the cell viability. For live/dead staining assay, cells were washed three times with PBS solution and 100 µL of DMEM containing 0.1% calcein-AM solution and 0.1% propidium iodide (PI) solution was added into each well. After incubation under dark conditions for 30 min, the stained L929 fibroblasts were observed under an inverted fluorescence microscope (DMi8, Leica).

### Antibacterial test

The antibacterial properties against *E. coli* and *S. aureus* of JPLCL/ZnO patch were evaluated by counting the number of colony-forming units (CFU) on broth agar plates. Prior to bacterial inoculation, all samples were sterilized using UV irradiation for 1 h. Colonies of *E. coli* and *S. aureus* were cultured in Luria-Bertani (LB) liquid medium and recovered by continuous shaking at 37 ℃ and 5% CO_2_ for 12 h. For antibacterial testing, JPLCL/ZnO patches (20 mm × 20 mm) were immersed in 2 mL of *E. coli* or *S. aureus* suspension (10^5^ CFU mL^−1^) and incubated at 37 °C for 12 h. In the experimental group, ultrasound treatment was applied at 0.5 W cm^−2^ for 5 min every 4 h. After incubation, the supernatants of each sample were diluted 10^6^-fold with PBS, and 100 µL of the diluted bacterial suspension was seeded on a broth agar plate and incubated for 18 h to assess the antibacterial efficacy. Digital images of the resulting bacterial colonies were captured for analysis. The antibacterial rate was calculated using the Eq. (1):

1 $$Antibacterial\, rate (\%)=(1-\:\frac{{C}_{t}}{{C}_{0}})\times100\%$$

where *C*_*0*_ is the concentration of the untreated bacterial solution, and *C*_*t*_ is the remaining concentration of bacteria after treatment in each experimental group.

### Cell migration test

Cell migration was evaluated using the cell scratch assay. L929 fibroblasts were seeded on 6-well plates at a density of 5 × 10^5^ cells per well and incubated in a humidified incubator with 5% CO_2_ at 37 °C. After cell adhesion, a scratch was created using a 200 µL sterilized pipette tip. Each well was then thoroughly washed with PBS to remove floating cells and filled with 2 mL of DMEM culture medium. In the experimental group, ultrasound treatment was applied at 0.5 W cm^−2^ for 5 min every 4 h. Images of the scratch were taken after treatment for 0, 8, 16, and 24 h using a fluorescence inverted microscope (AE31E, Motic, China). Scratch area was quantified using ImageJ software, and the cell migration rate was calculated as the ratio of the change in the scratch area over different time intervals to the initial scratch area using the Eq. (2):

2 $$Cell\, migration\, rate (\%)=\:\:\frac{{A}_{0}-{A}_{t}}{{A}_{0}}\:\times\:\:100\%$$

where *A*_*0*_ and *A*_*t*_ represent the areas of the initial scratch and the healed scratch area at different time intervals, respectively.

### Cell proliferation test

Cell proliferation was assessed by CCK-8 assay. PCO and JPLCL/ZnO patches were sterilized under ultraviolet light for 1 h. L929 fibroblasts were cocultured with PCO or JPLCL/ZnO patches at a density of 2.0 × 10^4^ cells per well with 1 mL of DMEM medium containing 10% FBS and 1% PS. In the experimental group, ultrasound treatment was applied once daily at 0.5 W cm^−2^ for 10 min. After 1, 2, and 3 days of incubation, the wells were washed three times with PBS, and 500 µL of DMEM containing 10% CCK-8 reagent was added to each well. The optical density was measured at a wavelength of 450 nm using a microplate reader (Varioskan LUX, Thermo Fisher).

### Cell adhesion test

Cell adhesion was tested to evaluate the effect of JPLCL/ZnO patch on cell behavior using cytoskeletal and nuclear staining. JPLCL/ZnO patches were cut to fit the wells of a 48-well plate and sterilized under ultraviolet light for 2 h. L929 fibroblasts were seeded at a density of 2 × 10^4^ cells per well on both the coated and uncoated surfaces of JPLCL/ZnO patch. After 24 h of incubation, the cells on both surfaces were washed three times with PBS and transferred to a new confocal dish for further staining. The cells were fixed with a 4% formaldehyde solution at room temperature for 10 min, followed by three washes with PBS. Permeabilization was performed using 0.5% Triton X-100 solution for 5 min. The cells were then stained with 500 µL of FITC-phalloidin (100 nM) and incubated in the dark at room temperature for 30 min. Nuclear counterstaining was carried out with 200 µL of Hoechst 33342 solution for 30 s. Finally, the stained cells were observed using a laser confocal microscope (ZEISS Axio Observer Z1).

### Macrophage polarization induction assay

To investigate the effect of JPLCL/ZnO patch on macrophage polarization, an inflammatory environment was first established by culturing Raw 264.7 macrophage cells with 100 ng mL^−1^ LPS for 12 h. PCO and JPLCL/ZnO patches were sterilized under ultraviolet light for 1 h. After sterilization, Raw 264.7 macrophage cells were cocultured with PCO or JPLCL/ZnO patches at a density of 2.0 × 10^4^ cells per well in 1 mL of DMEM medium containing 10% FBS and 1% PS. Ultrasound treatment was applied to JPLCL/ZnO at 0.5 W cm^−2^ for 5 min every 4 h. After 24 h of incubation, macrophage polarization was assessed using CD86 and CD206 markers to identify the M1 (pro-inflammatory) and M2 (anti-inflammatory) phenotypes, respectively. The polarization levels of macrophages in each group were evaluated using flow cytometry (FCM, CytoFLEX S) for quantification of M1/M2 ratio.

### In vivo biocompatibility

In vivo biocompatibility was evaluated by implanting PCO and JPLCL/ZnO patches into the dorsal subcutaneous tissue of rats. Adult male Sprague-Dawley rats (300–400 g) were used for in vivo studies. PCO and JPLCL/ZnO patches were cut into round shape with a diameter of approximately 1 cm and sterilized under ultraviolet light for 2 h. A subcutaneous incision was made on the dorsal area of the rats, where one to three implants were placed per rat. After 5 days, the animals were euthanized by CO_2_ inhalation. The excised subcutaneous tissues were then fixed in 4% paraformaldehyde for 24 h and sent to Wuhan Servicebio Technology Co., Ltd. for Hematoxylin-eosin (HE) staining and immunohistochemical staining.

### Abdominal wall defect repair experiment in rat

Animal surgery protocols for rat were reviewed and approved by the Animal Ethics Committee of Guangzhou Huateng Biomedical Technology Co., Ltd. (license number: C202310-3). Male Sprague-Dawley rats (300–400 g) were anesthetized with isoflurane (1%−2% isoflurane in oxygen) in an anesthetizing chamber. A circular defect (1.5 cm in diameter) was created in the middle of the abdominal wall using a standard biopsy punch, and the peritoneum along with full-thickness abdominal wall muscles was removed, leaving the skin intact. The defects were repaired by suturing the different patches into the defect site using 4-0 silk braided sutures. The rats were divided into five groups: (1) control group: defect without treatment; (2) US group: defect treated with ultrasound therapy alone; (3) PCO group: PCO patch applied to the defect; (4) JPLCL/ZnO group: JPLCL/ZnO patch applied to the defect; and (5) JPLCL/ZnO+US group: JPLCL/ZnO patch applied to the defect with ultrasound therapy. For the US and JPLCL/ZnO+US groups, ultrasound treatment (0.5 W cm^−2^ for 10 min per day) was applied for 6 days each week. After 14 days, the rats were euthanized by CO_2_ inhalation. The skin was dissected, and the sutured line was reopened to expose the repaired site for evaluation of adhesion formation and defect healing. The tissues were collected and sent to Wuhan Servicebio Technology Co., Ltd. for histological analysis, including HE staining, Masson staining, immunohistochemical staining, and immunofluorescence staining.

### Abdominal wall defect repair experiment in pig

Animal surgery protocols for pig were reviewed and approved by the A-Jentec Institutional Animal Care and Use Committee (license number: ZH20240910P). Female Landrace pigs (50 ± 5 kg) were anaesthetized with Zoletil 100 (2 mg kg^−1^) and 10% sodium chloride injection (4 mg kg^−1^) as a preanesthetic medication, followed by maintenance of anesthesia with isoflurane inhalation. A circular defect (2 cm in diameter and 1 cm in depth) was created on the inner side of the abdominal wall using an ultrasonic scalpel. The innermost abdominal wall muscle was excised, while the middle and outer muscle layers, as well as the skin, were preserved. The defect was then repaired by placing JPLCL/ZnO patches in either an aligned or misaligned direction relative to the anisotropic abdominal wall, using 4-0 silk braided sutures. The pigs were divided into three groups: (1) Mismatch group: JPLCL/ZnO patch applied to the defect with its orientation misaligned with the anisotropic direction of natural abdominal wall; (2) Mismatch+US group: JPLCL/ZnO patch applied to the defect with its orientation misaligned with the anisotropic direction of natural abdominal wall, combined with ultrasound therapy; (3) Match+US group: JPLCL/ZnO patch applied to the defect with its orientation aligned with the anisotropic direction of natural abdominal wall, combined with ultrasound therapy. In the Mismatch+US and Match+US groups, ultrasound treatment (0.5 W cm^−2^ for 10 min per day) was applied six times per week during the first two weeks. After 28 days, the pigs were euthanized by CO_2_ inhalation. Following skin dissection, the sutured area was reopened to expose the repaired site to evaluate adhesion formation and defect healing. The tissues were collected and sent to Wuhan Servicebio Technology Co., Ltd. for HE staining, Masson staining, immunohistochemical staining, and immunofluorescence staining.

### Statistical analysis

All statistical analysis was carried out using Origin 2021 software (Origin Lab Incorporation, Northampton, USA). The data were presented as mean ± standard deviation. Statistical analyses were performed using Student's t-test for comparisons between two groups and one-way analysis of variance (ANOVA) for multiple-group comparisons. Statistical significance was marked with * *P* < 0.05, ** *P* < 0.01, and *** *P* < 0.001.

## Results and discussion

**Fig. 2:**
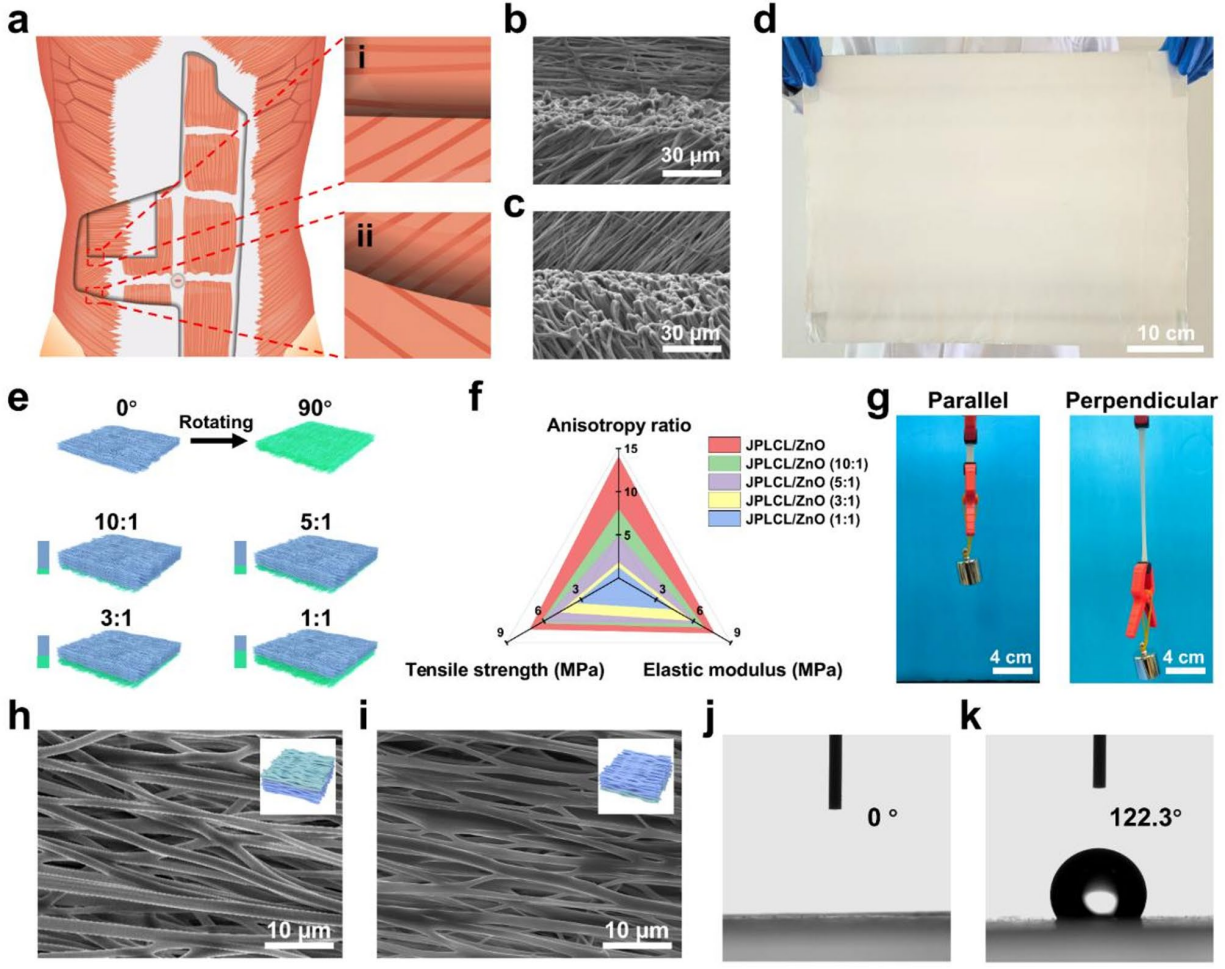
**a**) Schematic diagram of muscle fibers alignment in human abdominal wall: (**i**) The transversus abdominis (upper layer) exhibits muscle fibers aligned at 0°, while the internal oblique (lower layer) exhibits muscle fibers aligned at 45°. (**ii**) The internal oblique (upper layer) exhibits muscle fibers aligned at 45°, while the external oblique (lower layer) exhibits muscle fibers aligned at 135°. b, c) SEM images of a(**i**) region biomimetic JPLCL/ZnO patch (**b**) and a(**ii**) region biomimetic JPLCL/ZnO patch (**c**). **d**) Digital photo of large-scale JPLCL/ZnO patch. **e**) Schematic illustration of JPLCL/ZnO patches with different orientation stacking ratios. **f**) Quantitative analysis of anisotropy ratio, tensile strength, and elastic modulus of JPLCL/ZnO patches with different orientation stacking ratios. **g**) Digital photos of JPLCL/ZnO patch loaded with a 200 g weight in the parallel and perpendicular directions. **h**, **i**) SEM images of the top (**h**) and bottom (**i**) surfaces of JPLCL/ZnO patch. j, k) Water contact angles of the top (**j**) and bottom (**k**) surfaces of JPLCL/ZnO patch

PLCL was chosen for electrospinning due to its good flexibility, biodegradability, and biocompatibility [35, 36]. PLCL/ZnO patch was fabricated via multi-channel electrospinning of a polymer mixture containing PLCL, ZnO nanoparticles, and I-2959 photoinitiator. Subsequently, PLCL/ZnO patch was immersed in MPC solution to grow a PMPC coating on each fiber of the top surface by UV curing, yielding JPLCL/ZnO patch. Bioinspired by the anisotropic architecture of human abdominal wall, JPLCL/ZnO patch with a biomimetic muscle fiber-like morphology was fabricated by adjusting fiber orientation during electrospinning (Fig. 2a). As shown in Fig. 2b and c, our patch exhibits a morphology with fiber alignment similar to that of muscle fibers, with fibers aligned at 0°, 45°, and 135°, corresponding to the transversus abdominis, internal oblique, and external oblique muscles, respectively. Moreover, our patch can be produced at a large scale (Fig. 2d), demonstrating its potential for clinical translation. These results demonstrate that our patch with a biomimetic muscle fiber-like morphology has been successfully fabricated.

In clinical practice, designing patches that match the mechanical anisotropy of natural abdominal wall is crucial for defect repair, as mechanical mismatch can cause poor tissue integration, postoperative adhesion, and hernia recurrence [31, 34, 37]. Typically, patches used for the repair of abdominal wall defect in various regions are expected to possess region-specific anisotropy ratios (the mechanical property ratio in the coronal axis and vertical axis). To evaluate the mechanical anisotropy of our patches, stress-strain curves of JPLCL and JPLCL/ZnO patches were analyzed under tensile loading applied either parallel or perpendicular to the fiber orientation (Figure S1). Both patches exhibit significant anisotropy, with the parallel direction exhibiting significantly higher tensile strength and elastic modulus than the perpendicular direction (Figure S2). Specifically, compared with JPLCL patch, JPLCL/ZnO patch demonstrates a 61% increase in tensile strength (7.71 vs. 4.79 MPa) and 174% increase in elastic modulus (7.14 vs. 2.60 MPa) in the parallel direction (Figure S2). The enhanced mechanical properties are attributed to the incorporation of ZnO nanoparticles, which act as rigid nanofillers in the PLCL polymer matrix to improve load transfer, restrict polymer chain mobility, and thereby increase tensile strength [38–40]. To meet the mechanical anisotropy requirements of diverse regions of the abdominal wall, the mechanical properties of our JPLCL/ZnO patches can be changed according to the actual application requirements by adjusting the orientation stacking of the fibers. By adjusting the volume ratios of the polymer mixture electrospun in the 0° and 90° directions from 1:1 to 10:1 (Fig. 2e and Table S1), JPLCL/ZnO patches with different orientation stacking ratios (i.e., 10:1, 5:1, 3:1, and 1:1) were fabricated and their mechanical properties were investigated. Our JPLCL/ZnO patches can achieve anisotropy ratios ranging from approximately 1 to 14 (Fig. 2f and Figure S3), which can meet the full spectrum of abdominal wall anisotropy requirements (a range of 1–9) [29, 30]. Moreover, the tensile strength (4.39–7.71 MPa) and elastic modulus (3.49–7.14 MPa) of these patches meet or exceed the baseline requirements (0.08 MPa for tensile strength [31, 32] and 0.04 MPa for elastic modulus [41]) for abdominal wall defect repair (Fig. 2f). In addition to fiber orientation, ZnO content influences the mechanical performance of JPLCL/ZnO patches. As shown in Figure S4, JPLCL/ZnO patches with higher ZnO contents exhibit increased tensile strength and elastic modulus, indicating the reinforcing role of ZnO nanoparticles in the PLCL matrix. Considering the anisotropic gradient distribution characteristics of human abdominal wall, we have selected JPLCL/ZnO patch with 5 wt% ZnO and the highest anisotropy as the representative sample for our study to investigate the effects of structural anisotropy on mechanical performance and tissue regeneration. Under a 200 g load, JPLCL/ZnO patch exhibits greater elongation in the perpendicular direction than in the parallel direction (Fig. 2g), demonstrating its high flexibility and mechanical anisotropy. Therefore, by adjusting the orientation stacking of the fibers, JPLCL/ZnO patches possess tunable anisotropy ratios, tensile strength, and elastic modulus, allowing them to adaptively match the mechanical requirements of natural abdominal wall.

The fiber alignment of porcine abdominal wall muscle was evaluated through optical microscope. As shown in Figure S5, the porcine abdominal wall exhibits an aligned fibrous architecture with visible fiber bundles. To further assess fiber morphology, tissue samples were harvested and sectioned in two directions for HE staining: longitudinal section (parallel to the muscle fiber direction) and cross section (perpendicular to the fiber direction). As shown in Figure S6, the muscle fibers are highly aligned with an average diameter of approximately 50 μm, which is consistent with reported diameters of porcine muscle fibers with a range of 47–98 μm [42, 43]. Inspired by the anisotropic architecture of the abdominal wall, we have fabricated JPLCL and JPLCL/ZnO patches to mimic its native structure for defect repair. The morphologies of these patches were characterized by scanning electron microscopy (SEM). JPLCL and JPLCL/ZnO patches exhibit fibrous morphologies, with an increased fiber diameter on the top surface (PMPC-coated surface) compared to the bottom surface following the deposition of the PMPC coating (Fig. 2h and i). Orientation analysis further reveals that most fibers are aligned within a narrow deviation of −20° to 20° (Figures S7 and S8), indicating the highly oriented fibrous structure. The fiber orientation observed in our JPLCL/ZnO patch closely mimics the highly aligned structure of porcine abdominal wall muscle, which is crucial for mimicking the anisotropic characteristics of natural abdominal wall in the application of defect repair.

**Fig. 3:**
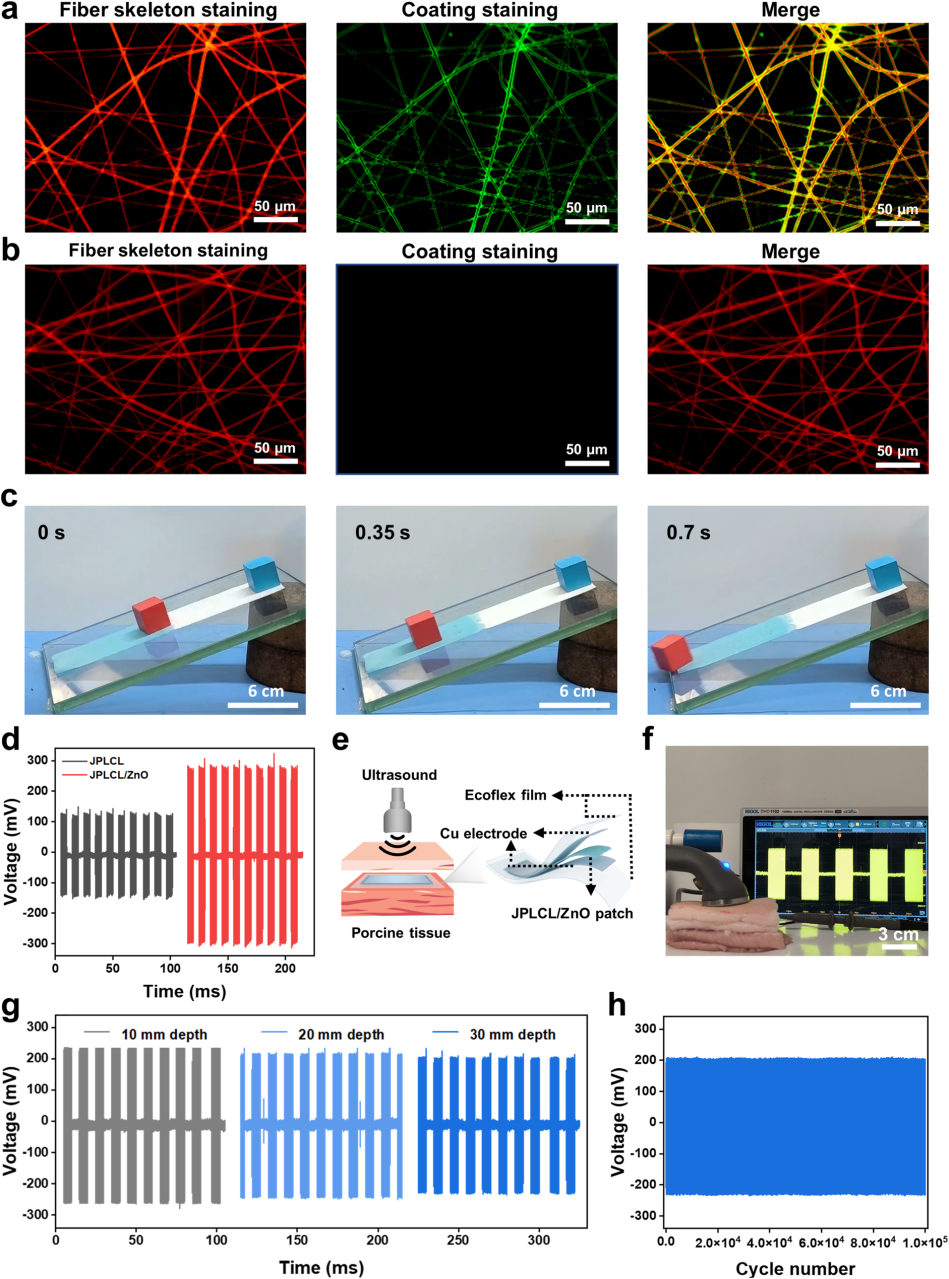
**a**, **b**) Fluorescence microscopy images of the top (**a**) and bottom (**b**) surfaces of JPLCL/ZnO patch. **c**) Digital photos of the slope slide experiment by sliding two ceramic blocks on the top (blue color) and bottom (white color) surfaces of JPLCL/ZnO patch. **d**) Output voltages of JPLCL and JPLCL/ZnO patches under ultrasound stimulation. **e**, **f**) Schematic illustration (**e**) and digital photo (**f**) of voltage generation of JPLCL/ZnO patch in porcine tissue under ultrasound stimulation. **g**) Output voltages of JPLCL/ZnO patch implanted at different depths of porcine tissue. **h**) Cyclic stability of the output voltage of JPLCL/ZnO patch implanted at a 30 mm depth of porcine tissue during 10^5^ cycles

The Janus structure of our JPLCL/ZnO patches was characterized using X-ray photoelectron spectroscopy (XPS) and water contact angle measurements. XPS spectra confirm the presence of P and N elements on the top surface of JPLCL/ZnO patch (Figure S9), indicating the successful formation of PMPC. As shown in Fig. 2j and k, the water contact angle is almost 0° on the top surface and 122° on the bottom surface of JPLCL/ZnO patch, indicating the superhydrophilic nature of PMPC coating. This highly hydrated surface can effectively resist protein adsorption and cellular attachment, thereby preventing tissue adhesion in defect repair [44, 45]. Moreover, JPLCL/ZnO patch exhibits a fibrous morphology with a rough surface, as indicated by the C, O, and P elements predominantly distributed along the fibers (Figure S10), indicating that PMPC coating does not disrupt the original fibrous morphology of JPLCL/ZnO patch. In contrast, we prepared a PLCL/ZnO patch with Janus structure using traditional benzophenone treatment (BPLCL/ZnO). It can be clearly seen from the SEM image (Figure S11) that the original fibrous morphology of BPLCL/ZnO patches is covered by a continuous PMPC coating, with C, O, and P elements uniformly distributed across the entire surface. Traditional benzophenone treatment could destroy the highly oriented morphology and mechanical anisotropy of the patches [44], leading to a mechanical mismatch with the abdominal wall and hindering the repair process. To better visualize the spatial relationship between fibers and coatings, JPLCL/ZnO patch was stained with Nile red for fiber skeleton and sodium fluorescein for PMPC coating, and subsequently observed under a fluorescence microscope. Red-stained fiber skeleton and green-stained coating are clearly visible on the top surface of JPLCL/ZnO patch, with the green-stained coating seamlessly conforming to the red-stained fiber skeleton (Fig. 3a), demonstrating that PMPC coating is formed on each fiber of the top surface. In contrast, only the red-stained fiber skeleton is observed on the bottom surface (Fig. 3b), further confirming the Janus structure of JPLCL/ZnO patch. As a conceptual characterization, we conducted a slope slide experiment by sliding two ceramic blocks over both surfaces of JPLCL/ZnO patch. The top surface of JPLCL/ZnO patch was stained blue with a 0.01% (w/v) erioglaucine disodium salt solution (Brilliant Blue FCF, an FDA-approved biocompatible dye [46, 47]) to distinguish it from the white bottom surface. As shown in Fig. 3c, the red ceramic block moves smoothly on the top surface, while no visible movement of the blue ceramic block is observed on the bottom surface (Video S1), indicating the excellent lubricating properties of PMPC coating. These results demonstrate that JPLCL/ZnO patch with a Janus structure has been successfully fabricated by constructing PMPC coating on each fiber on its top surface.

Wireless electrostimulation therapy, such as ultrasound therapy, has shown great potential in promoting tissue repair due to its non-invasive nature and precise targeting capability [48, 49]. Piezoelectric materials can be wirelessly activated by ultrasound to produce localized electrical stimulation to promote cell behavior and tissue regeneration [50, 51]. The electric performances of JPLCL/ZnO patches with different ZnO contents were evaluated under ultrasound stimulation at a power intensity of 0.5 W cm^−2^ and a pulse duty of 50%. As shown in Figure S12, the output voltage of JPLCL/ZnO patch increases progressively with ZnO content. In contrast to JPLCL patch, JPLCL/ZnO patch exhibits an ~ 2.4-fold increase in output voltage (Fig. 3d), demonstrating the enhanced electrical performance with the incorporation of ZnO nanoparticles. Under ultrasound stimulation, our patch undergoes cyclic deformation that induces electrical polarization and produces localized electric signals, which have been shown to regulate cellular behaviors such as cell migration and proliferation [52–54]. Considering that the thickness of human abdominal wall typically ranges from 10 to 30 mm [55], an ex vivo implantation model was designed to simulate its clinical application. As shown in Fig. 3e, JPLCL/ZnO patch was encapsulated between two copper (Cu) electrodes and Ecoflex films to construct a flexible device, which was then implanted at different depths of porcine tissue. An ultrasound probe was used to deliver ultrasound stimulation through the porcine skin, and the output voltages of JPLCL/ZnO patch were measured and recorded using an oscilloscope (Fig. 3f). As the implantation depth in porcine tissue increases from 10 mm to 30 mm, the output voltages of JPLCL/ZnO patch show a slight decrease (Fig. 3g and Figure S13). Moreover, our JPLCL/ZnO patch maintains stable electrical output even at a depth of 30 mm, with no significant voltage differences between the initial and final cycles over 10^5^ cycles (Fig. 3h), indicating that ultrasound therapy can effectively penetrate tissues and precisely focus on targeted areas in a non-invasive manner. Therefore, our JPLCL/ZnO patch can be wirelessly activated by ultrasound to generate localized electrical stimulation for defect repair.

Good biocompatibility and antibacterial properties are essential for implanted materials in tissue repair to ensure both biosafety and prevent infection [56–59]. The biocompatibility of JPLCL/ZnO patch was evaluated using live/dead staining and cell counting kit-8 (CCK-8) assays. The morphologies and cell density of the JPLCL/ZnO group are similar to those of the control group (Figure S14a). In addition, the quantitative analysis of CCK-8 assay shows no significant statistical difference in cell proliferation between the two groups (Figure S14b), demonstrating the good biocompatibility of JPLCL/ZnO patch. In vivo biocompatibility was further evaluated by implanting PCO and JPLCL/ZnO patches subcutaneously in the dorsal region of rats for 5 days, after which tissue samples were collected for HE and immunohistochemical staining, including CD68 (a macrophage marker) and IL-6 (an inflammatory factor). The JPLCL/ZnO group shows a lower inflammatory response compared to the PCO group, and the expressions of CD68 and IL-6 in the JPLCL/ZnO group are significantly lower than those in the PCO group (Figure S15), indicating that JPLCL/ZnO patch does not induce obvious inflammatory reaction. To further evaluate the in vivo biosafety of the patches under ultrasound stimulation, major organs (e.g., heart, liver, spleen, lung, and kidney) were collected for HE staining after rats were sacrificed. As shown in Figure S16, no evident tissue damage is observed in the organs, indicating that our patch under ultrasound therapy exhibits good biosafety in vivo. In addition, the antibacterial properties of JPLCL/ZnO patch against *E. coli* and *S. aureus* were evaluated using the agar plate incubation method. The JPLCL/ZnO+US group shows fewer colony-forming units (CFU) of *E. coli* and *S. aureus* compared to the control and JPLCL/ZnO groups (Figure S17a). JPLCL/ZnO patch shows bacteriostatic rates of 88% and 86% against *E. coli* and *S. aureus*, respectively, which are further enhanced to 94% and 93% under ultrasound stimulation (Figures S17b and S17c), suggesting a synergistic antibacterial effect between the patch and ultrasound therapy. These results demonstrate that JPLCL/ZnO patch exhibits good biocompatibility and antibacterial properties.

**Fig. 4:**
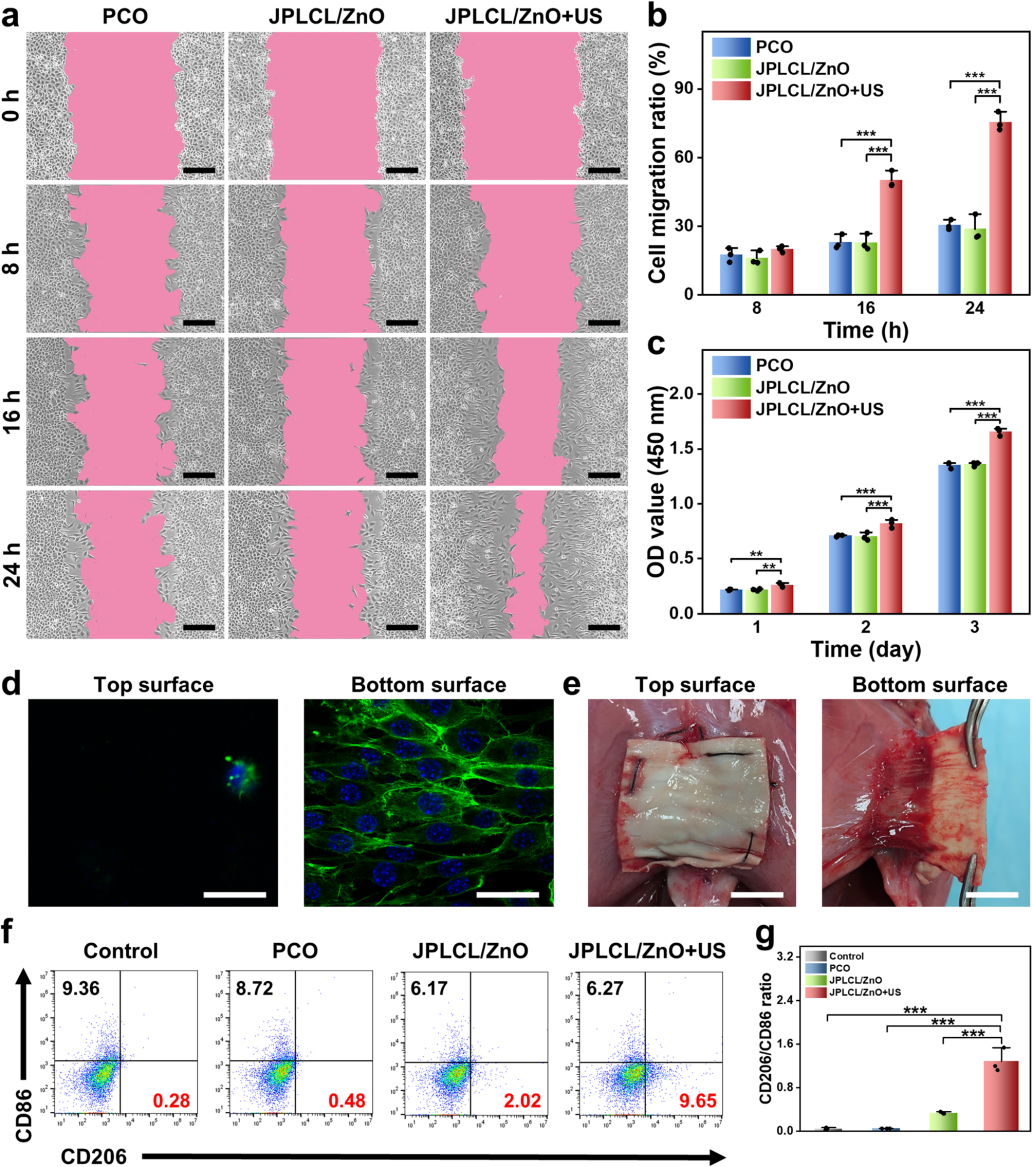
**a**) Cell migration images of fibroblasts for 0, 8, 16, and 24 h in the PCO, JPLCL/ZnO, and JPLCL/ZnO + US groups (scale bars: 200 μm). **b**) Quantitative analysis of cell migration (*n* = 3 independent samples; ANOVA followed by Tukey’s multiple comparisons; *** adjusted *P* < 0.001; error bars = SD; data are presented as mean ± SD). **c**) CCK-8 assay of L929 fibroblasts cultured after 1, 2, and 3 days in the PCO, JPLCL/ZnO, and JPLCL/ZnO + US groups (*n* = 3 independent samples; ANOVA followed by Tukey’s multiple comparisons; ** adjusted *P* < 0.01; *** adjusted *P* < 0.001; error bars = SD; data are presented as mean ± SD). **d**) Fluorescence images of L929 fibroblasts cultured on the top and bottom surfaces of JPLCL/ZnO patch for 1 day (scale bars: 40 μm). **e**) Digital photos of soft tissue adhering to JPLCL/ZnO patch in repairing a rat abdominal wall defect on the 14th day after surgery (scale bars: 1 cm). **f**) Quantification of CD206 and CD86 on RAW 264.7 cells by flow cytometry. **g**) Quantitative analysis of CD206/CD86 ratio (*n* = 3 independent samples; ANOVA followed by Tukey’s multiple comparisons; *** adjusted *P* < 0.001; error bars = SD; data are presented as mean ± SD)

The migration and proliferation of fibroblasts are crucial indicators in the tissue repair process, as they produce various extracellular matrix components and cytokines to promote tissue regeneration [60–63]. To investigate the activity of fibroblasts cultured with the patch under ultrasound stimulation, cell migration and proliferation rates were evaluated by cell scratch test and cell proliferation assay. The in vitro cell scratch test results show that the JPLCL/ZnO+US group significantly promotes the migration of fibroblasts compared to the control and JPLCL/ZnO groups (Fig. 4a and b), which can be attributed to efficient guiding effect of electric fields under ultrasound therapy. Cell proliferation rate was quantitatively evaluated using CCK-8 assay. L929 fibroblasts in the JPLCL/ZnO+US group exhibit a higher cell count than those in the control and JPLCL/ZnO groups (Fig. 4c), demonstrating its ability to promote cell proliferation. In addition to promoting cell migration and proliferation, abdominal wall defect repair materials also need asymmetric regulation of cells to achieve anti-adhesion performance. Therefore, the cell adhesion behaviors of JPLCL/ZnO patches were assessed to evaluate the effect of the Janus structure on biological adhesion. L929 fibroblasts were seeded on both the top and bottom surfaces of JPLCL/ZnO patch and cultured for one day to capture the fluorescence staining images. As shown in Fig. 4d, more L929 fibroblasts adhere to the bottom surface of JPLCL/ZnO patch and grow along the anisotropic fibers, while only few L929 fibroblasts are observed on the top surface, indicating that our JPLCL/ZnO patch can promote directional cell growth while preventing tissue adhesion. In contrast, a random JPLCL/ZnO patch was fabricated at a drum rotation speed of 100 rpm, and L929 fibroblasts cultured on these random fibers exhibit multidirectional growth and attachment due to the lack of anisotropic topology (Figure S18). The bioadhesion behavior of JPLCL/ZnO patch was further evaluated in vivo using a rat abdominal wall defect model. After 14 days of implantation, the bottom surface of JPLCL/ZnO patch adheres well to the defect tissue, while its top surface can effectively prevent visceral adhesion (Fig. 4e). These results demonstrate that our JPLCL/ZnO patch can not only promote cell migration and proliferation, but also prevent visceral adhesion.

Except for cell migration and proliferation, macrophage polarization plays a crucial role in tissue repair by regulating inflammation and promoting healing [64–66]. To investigate the effect of JPLCL/ZnO patch on immunoregulation, the expression of CD86 (a marker of M1 macrophages) and CD206 (a marker of M2 macrophages) were evaluated using flow cytometry. As shown in Fig. 4f and g, the JPLCL/ZnO+US group exhibits the highest M2-like/M1-like macrophage ratio among all the groups, indicating that JPLCL/ZnO patch under ultrasound therapy can enhance M2 macrophage polarization.

**Fig. 5:**
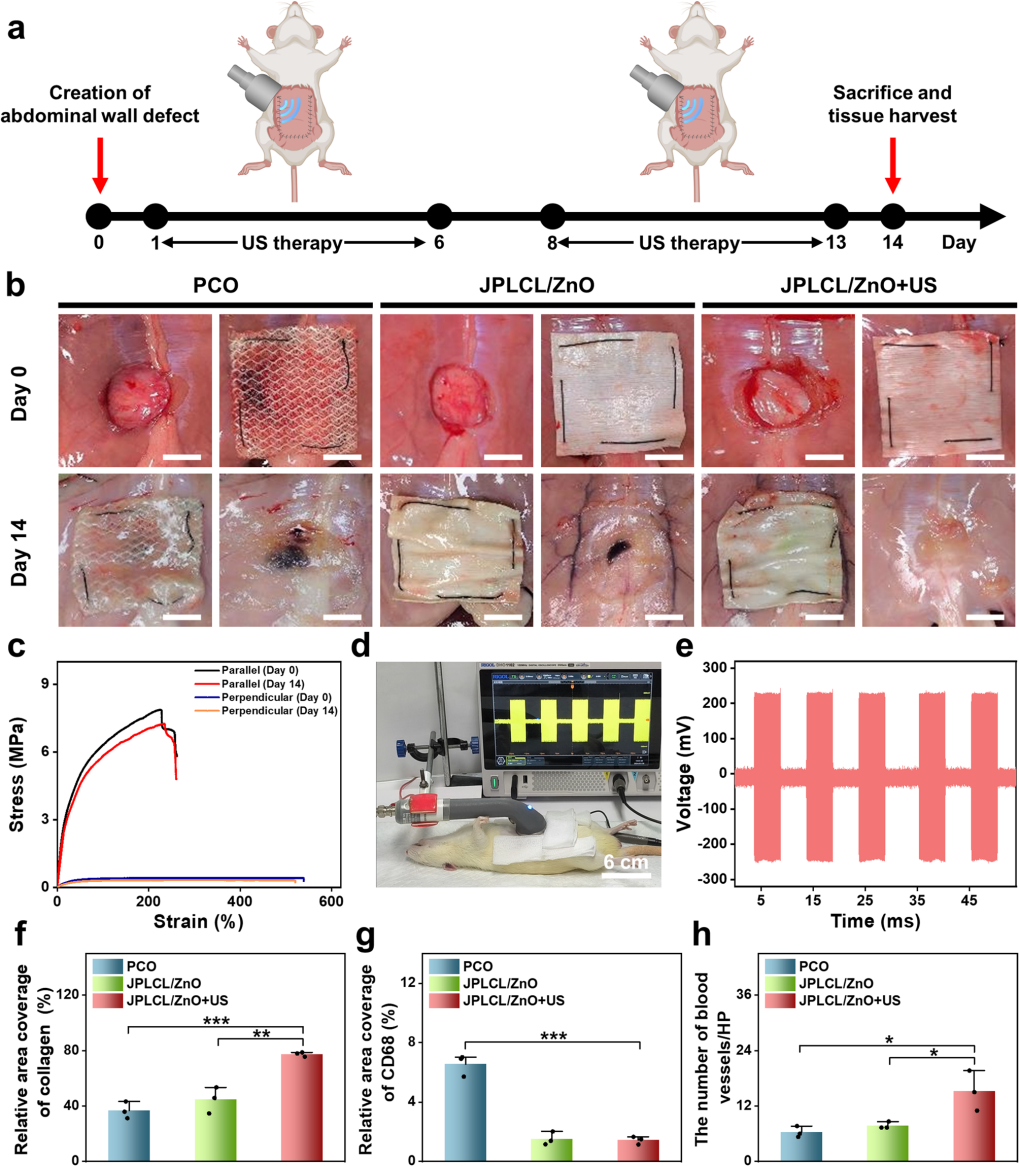
**a**) Schematic diagram of the repair of abdominal wall defect in a rat model. **b**) Digital photos of wound healing and visceral adhesions formation of abdominal wall defects in the PCO, JPLCL/ZnO, and JPLCL/ZnO + US groups. Day 0: surgically created defect (left) and sutured patch covering the defect (right). Day 14: implanted patch in situ (left) and defect site after patch removal (right) (scale bars: 1 cm). **c**) Mechanical properties of JPLCL/ZnO patches in the parallel and perpendicular directions before implantation and after 14 days of in vivo implantation. **d**) Digital photo of voltage generation from JPLCL/ZnO patch under ultrasound stimulation in a rat model. **e**) Output voltage of JPLCL/ZnO patch under ultrasound stimulation. **f**) Quantitative analysis of collagen density (*n* = 3 independent samples; ANOVA followed by Tukey’s multiple comparisons; ** adjusted *P* = 0.002; *** adjusted *P* < 0.001; error bars = SD; data are presented as mean ± SD). **g**) Quantitative analysis of CD68 (*n* = 3 independent samples; ANOVA followed by Tukey’s multiple comparisons; *** adjusted *P* < 0.001; error bars = SD; data are presented as mean ± SD). **h**) Quantitative analysis of CD31/α-SMA (*n* = 3 independent samples; ANOVA followed by Tukey’s multiple comparisons; PCO vs. JPLCL/ZnO + US * adjusted *P* = 0.014; JPLCL/ZnO vs. JPLCL/ZnO + US * adjusted *P* = 0.031; error bars = SD; data are presented as mean ± SD)

To evaluate the in vivo pro-healing and anti-adhesion properties of JPLCL/ZnO patch, a rat abdominal wall defect model was established by creating a 15 mm circular defect in the abdominal wall using a circular punch. On the 14th day post-surgery, rats were sacrificed to observe defect repair and adhesion formation (Fig. 5a). Representative photos of wound healing and visceral adhesion are shown in Fig. 5b, including the surgically created defect and sutured patch on day 0, and the implanted patch in situ and the defect site after patch removal on day 14. JPLCL/ZnO patch does not cause severe visceral adhesion, and its anti-adhesion effect is comparable to that of clinical PCO patch. After removing the patches to expose the defects, the JPLCL/ZnO+US group exhibits markedly increased healing compared with the other groups (Fig. 5b). To evaluate the mechanical properties of JPLCL/ZnO patch in vivo, stress-strain curves were analyzed before implantation and 14 days after implantation. JPLCL/ZnO patch shows excellent mechanical anisotropy on the 14th day (Fig. 5c), which is beneficial for defect repair and long-term implantation [31, 67, 68]. In addition, an in vivo electrical performance experiment was conducted to investigate the feasibility of voltage generation from the patch under ultrasound stimulation in a rat model. After anesthetizing the rat with 3% pentobarbital sodium via intraperitoneal injection, JPLCL/ZnO patch was implanted in the abdominal wall defect and then exposed to ultrasound waves, the output voltage was recorded using an oscilloscope (Fig. 5d and Video S2). Under an ultrasound power intensity of 0.5 W cm^−2^, our JPLCL/ZnO patch can generate an output voltage of 231 mV (Fig. 5e), confirming the feasibility of delivering electric stimulation to the defect under ultrasound therapy.

To further evaluate the pro-healing properties of JPLCL/ZnO patches, tissue samples were collected from the rat abdominal wall defects on the 14th day after surgery for histological analysis. As shown in Figure S19, HE staining results indicate that both the control and US groups exhibit incomplete healing, indicating that ultrasound therapy alone hardly promotes wound healing. In contrast to the PCO and JPLCL/ZnO groups, the JPLCL/ZnO + US group shows reduced infiltration of inflammatory cells and denser collagen bundles (Figures S20 and S21). Quantitative analysis reveals that the collagen density in the JPLCL/ZnO+US group (77.4%) is significantly higher than the PCO (36.8%) and JPLCL/ZnO (44.7%) groups (Fig. 5f), indicating its excellent pro-healing property. Moreover, immunohistochemical staining results reveal that the expression of CD68 is significantly lower in both the JPLCL/ZnO and JPLCL/ZnO+US groups compared to the PCO group (Fig. 5g and Figure S22). Double immunofluorescence staining for CD31 and α-SMA was performed to evaluate angiogenesis. CD31 serves as a marker of endothelial cells and reflects new blood vessel formation, while α-SMA is a marker of vascular smooth muscle cells and indicates vessel maturation. The expression levels of CD31 and α-SMA in the JPLCL/ZnO+US group are significantly higher than those in the PCO and JPLCL/ZnO groups (Fig. 5h and Figure S23), indicating that JPLCL/ZnO patch can promote vascular proliferation under ultrasound stimulation. These results provide reliable histological evidence supporting the pro-healing ability of our JPLCL/ZnO patch under ultrasound therapy.

**Fig. 6:**
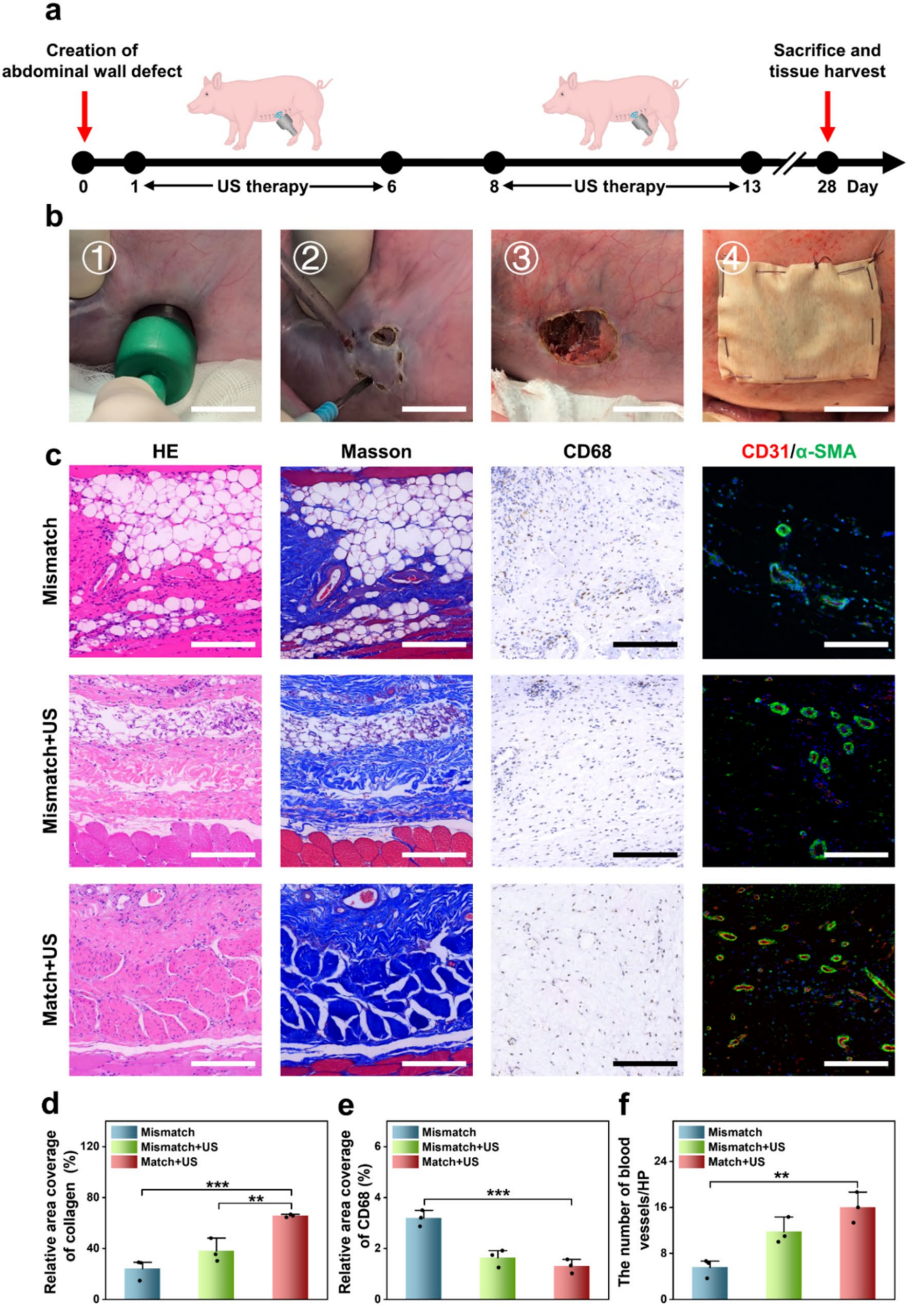
**a**, **b**) Schematic diagram (**a**) and surgical process (**b**) of the repair of abdominal wall defect in a porcine model (scale bars: 2 cm). **c**) Images of HE staining, Masson staining, immunohistochemical staining, and immunofluorescence staining for the Mismatch, Mismatch+US, and Match+US groups (scale bars: 200 μm) **d**) Quantitative analysis of collagen density (*n* = 3 independent samples; ANOVA followed by Tukey’s multiple comparisons; ** adjusted *P* = 0.008; *** adjusted *P* < 0.001; error bars = SD; data are presented as mean ± SD). **e**) Quantitative analysis of CD68 (*n* = 3 independent samples; ANOVA followed by Tukey’s multiple comparisons; *** adjusted *P* < 0.001; error bars = SD; data are presented as mean ± SD). **f**) Quantitative analysis of CD31/α-SMA (*n* = 3 independent samples; ANOVA followed by Tukey’s multiple comparisons; ** adjusted *P* =0.003; error bars = SD; data are presented as mean ± SD)

To simulate a clinically relevant abdominal wall defect in human, a porcine abdominal wall defect model was established by creating a 2 cm circular defect in the abdominal wall using an ultrasonic scalpel. These defects were subsequently repaired using patches, which were secured with 4-0 silk braided sutures **(**Fig. 6a and b). The experimental treatments were divided into three groups: the Mismatch group receiving a JPLCL/ZnO patch that misaligned with the natural abdominal wall anisotropy direction, the Mismatch+US group receiving a JPLCL/ZnO patch misaligned with the natural abdominal wall anisotropy direction and ultrasound therapy, and the Match+US group receiving a JPLCL/ZnO patch aligned with the natural abdominal wall anisotropy direction and ultrasound therapy. For the Mismatch+US and Match+US groups, ultrasound treatment was administered at 0.5 W cm^−2^ for 10 min per day and applied six days per week. After 28 days, the pigs were euthanized and tissue samples were harvested for histological analysis. HE and Masson staining results indicate that the Match+US group exhibits superior tissue alignment and increased collagen deposition compared to the Mismatch and Mismatch+US groups (Fig. 6c and d). This suggests that the biomechanical matching and ultrasound therapy can synergistically promote the repair of abdominal wall defects. Immunohistochemical and immunofluorescence staining further reveal that the Match + US group shows lower expression of CD68 and higher expression of CD31 and α-SMA compared to the Mismatch and Mismatch+US groups (Fig. 6c and e, and 6f). These results demonstrate that our JPLCL/ZnO patch can effectively promote tissue alignment, collagen deposition, and vascular proliferation under ultrasound therapy. Therefore, our JPLCL/ZnO patch not only activates electrical stimulation to promote tissue repair under ultrasound therapy, but also integrates excellent mechanical anisotropy and anti-adhesion properties for the efficient repair of abdominal wall defects.

## Conclusion

In conclusion, we have successfully developed an attractive ultrasound-responsive JPLCL/ZnO patch through multi-channel electrospinning and in-situ photocuring strategies. Our JPLCL/ZnO patch has tunable mechanical anisotropy by regulating fiber orientation stacking ratio, thus achieving biomechanical match with natural abdominal wall tissue. Meanwhile, JPLCL/ZnO patch produces localized electrical stimulation to promote cell migration and proliferation owing to wireless activation via external ultrasound. Furthermore, the in-situ coating of PMPC on the top surface endows our JPLCL/ZnO patch with excellent anti-adhesion performance. As a result, our JPLCL/ZnO patch demonstrates outstanding mechanical anisotropy, pro-healing, and anti-adhesion properties for abdominal wall defect repair in the rat and porcine models. Our work could offer a promising strategy for dynamic soft tissue repair.

## Supplementary Information

Supplementary Material 1

Supplementary Material 2

Supplementary Material 3

## Data Availability

No datasets were generated or analysed during the current study.
