# Supplementary material for: Ultrasound-responsive Janus patch with mechanical anisotropy, pro-healing, and anti-adhesion properties for abdominal wall defect repair
Source: J Nanobiotechnology. 2025 Nov 5;23:702. doi: 10.1186/s12951-025-03779-z (PMC12587614; doi:10.1186/s12951-025-03779-z)
Supplement: Supplementary file 3 — Supplementary Material 3 [file 12951_2025_3779_MOESM3_ESM.docx]

**Supporting Information**

**Ultrasound-responsive Janus patch with mechanical** **anisotropy, pro-healing, and anti-adhesion properties for abdominal wall defect repair**

Binying Peng^a,1^, Binghua Ma^c,1^, Hao Lu^d,1^, Zixin Chen^a,1^, Wenxuan Xiong^a^, Hui Wang^a,*^, Zhaopeng Cai^b,*^, Xingxing Shi^b,*^, Rongkang Huang^a,*^

^a^ Department of General Surgery (Colorectal Surgery), Guangdong Institute of Gastroenterology, Biomedical Innovation Center, Key Laboratory of Human Microbiome and Chronic Diseases (Sun Yat-sen University), Ministry of Education, Guangdong Provincial Key Laboratory of Colorectal and Pelvic Floor Diseases, The Sixth Affiliated Hospital, Sun Yat-sen University, Guangzhou 510655, P. R. China

^b^ The Eighth Affiliated Hospital, Sun Yat-sen University, Shenzhen 518033, P. R. China

^c^ Translational Medicine Research Center, Naval Medical University, Shanghai 200433, P. R. China

^d^ Department of Colorectal Surgery, Second Affiliated Hospital of Navy Medical University, Shanghai 200003, P. R. China

^*^Corresponding author.

*E-mail addresses*: huangrk3@mail.sysu.edu.cn (R. Huang), shixx7@mail.sysu.edu.cn (X. Shi), caizhp3@mail.sysu.edu.cn (Z. Cai), wang89@mail.sysu.edu.cn (H. Wang).

^1^ These authors contributed equally to the work.


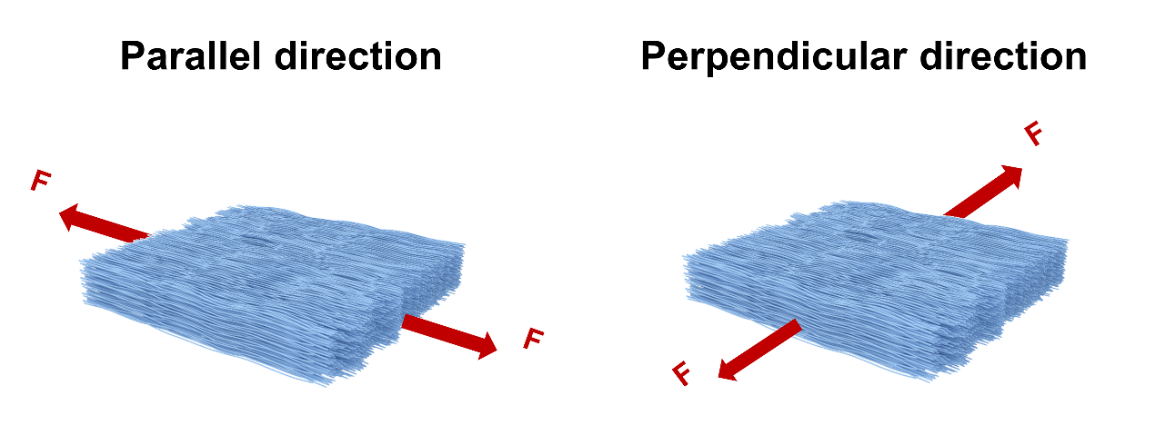


**Figure S1.** Schematic illustration of the tensile test showing tensile loading applied either parallel or perpendicular to the fiber orientation.


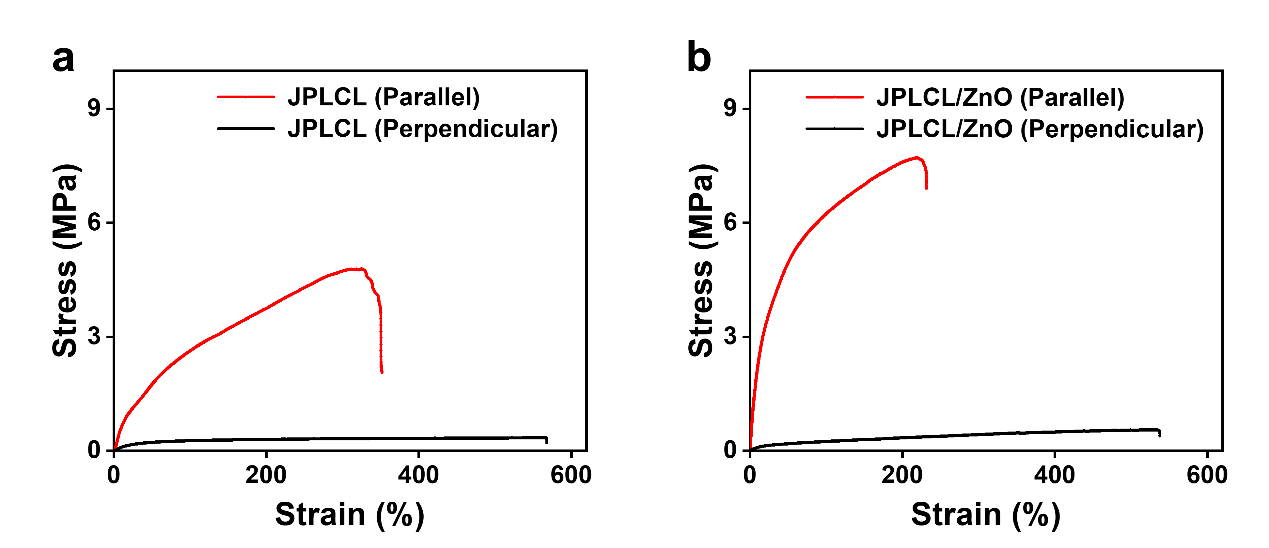


**Figure S2.** Stress-strain curves of JPLCL (a) and JPLCL/ZnO (b) patches in the parallel and perpendicular directions.


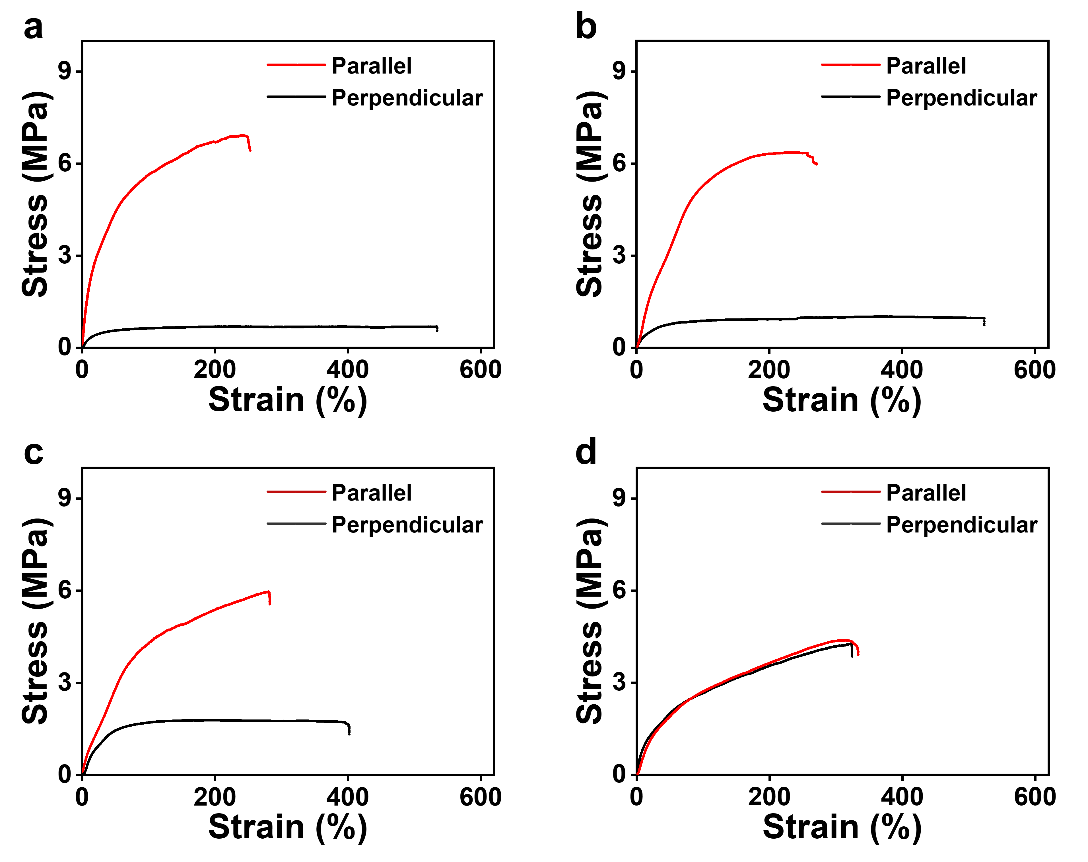


**Figure S3.** Stress-strain curves of JPLCL/ZnO patches with different orientation stacking ratios: 10:1 (a), 5:1 (b), 3:1 (c), and 1:1 (d).





**Figure S4.** Stress-strain curves of JPLCL/ZnO patches with different ZnO contents in the parallel direction.


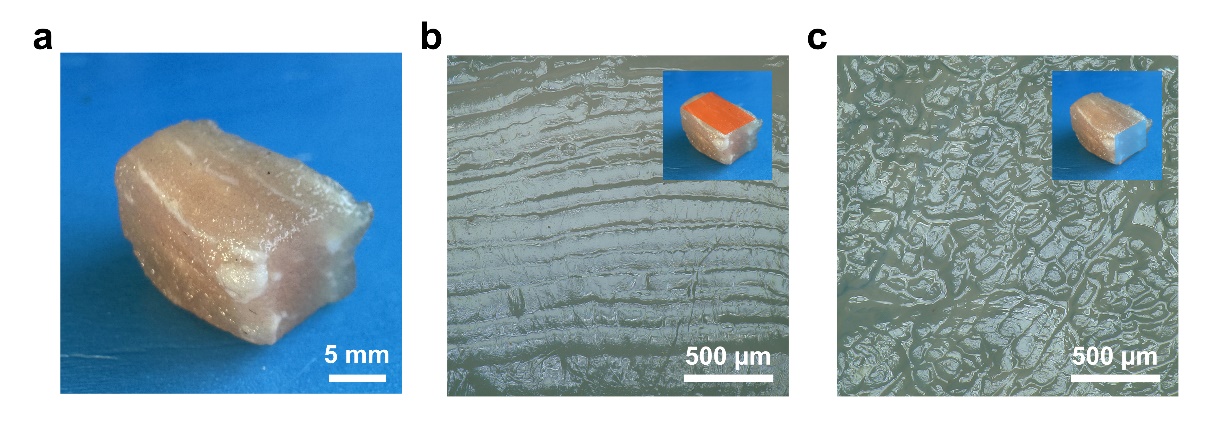


**Figure S5.** a) Digital photo of the porcine abdominal wall muscle. b, c) Microscope images of the top view (red color) (b) and side view (blue color) (c) of porcine abdominal wall muscle fibers.


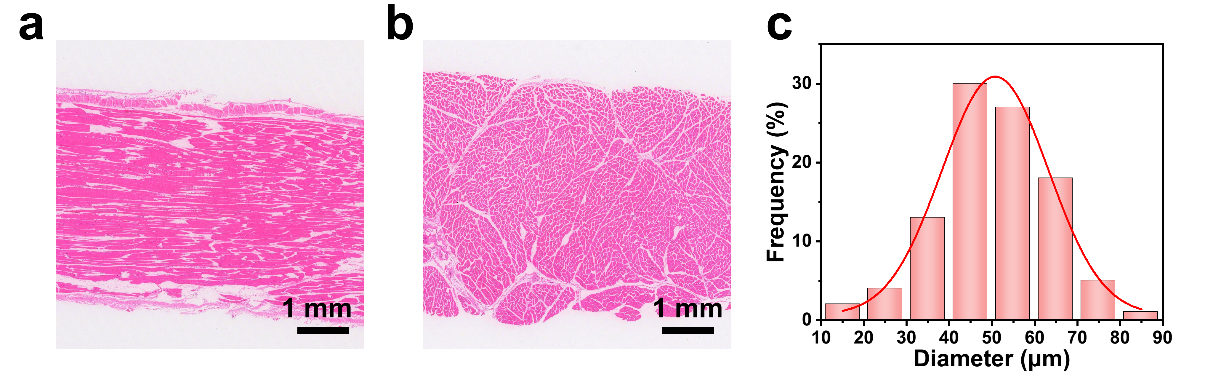


**Figure S6.** a, b) Images of HE staining of porcine abdominal muscle fibers in the longitudinal section (a) and cross section (b). c) Diameter distributions of muscle fibers.


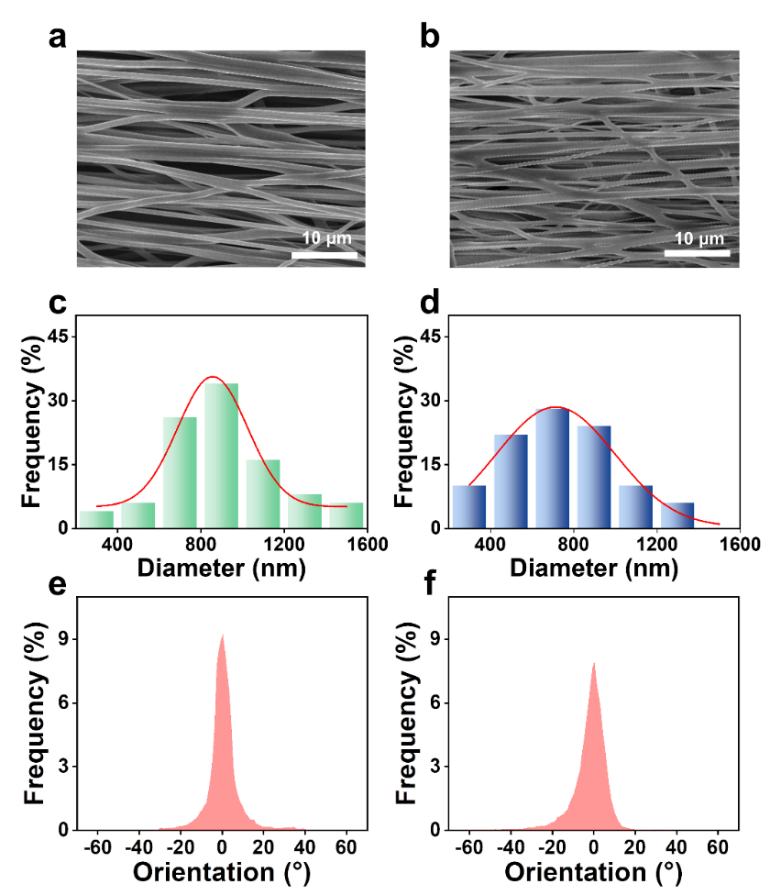


**Figure S7.** a, b) SEM images of the top (a) and bottom (b) surfaces of JPLCL patch. c, d) Fiber diameter distributions of the top (c) and bottom (d) surfaces of JPLCL patch. e, f) Orientation distributions of the top (e) and bottom (f) surfaces of JPLCL patch.


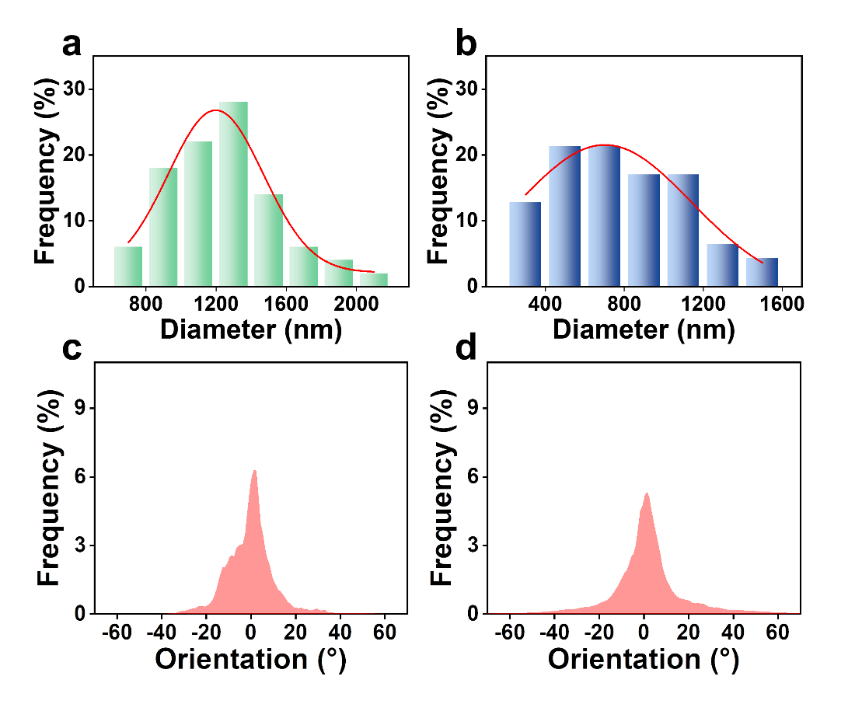


**Figure S8.** a, b) Fiber diameter distributions of the top (a) and bottom (b) surfaces of JPLCL/ZnO patch. c, d) Orientation distributions of the top (c) and bottom (d) surfaces of JPLCL/ZnO patch.


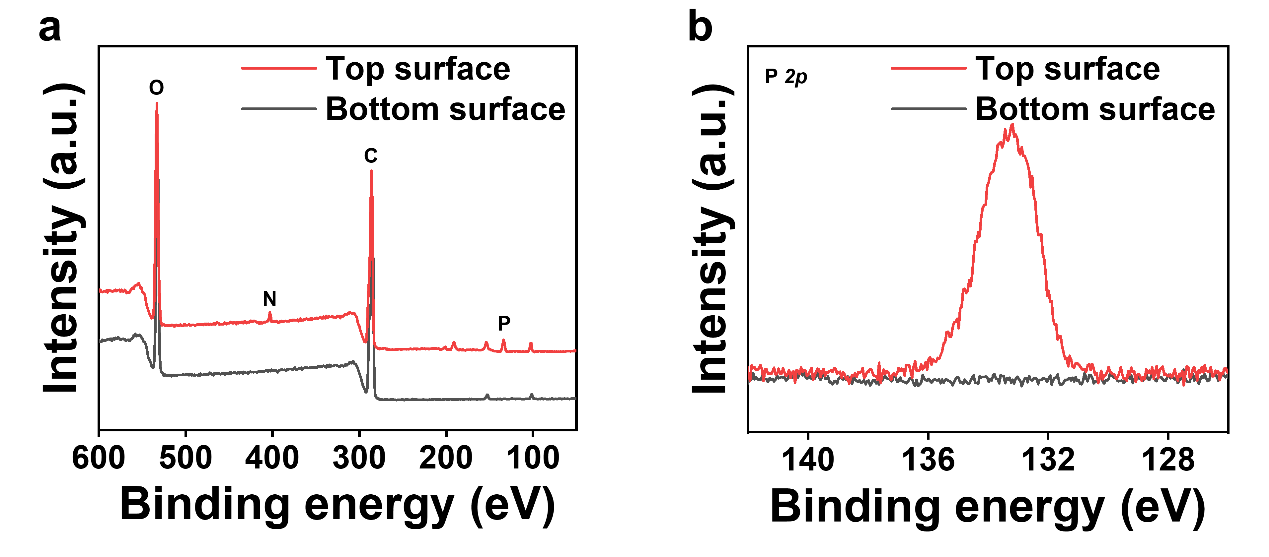


**Figure S9.** XPS spectra (a) and high-resolution XPS spectra of P 2p (b) of the top and bottom surfaces of JPLCL/ZnO patch.


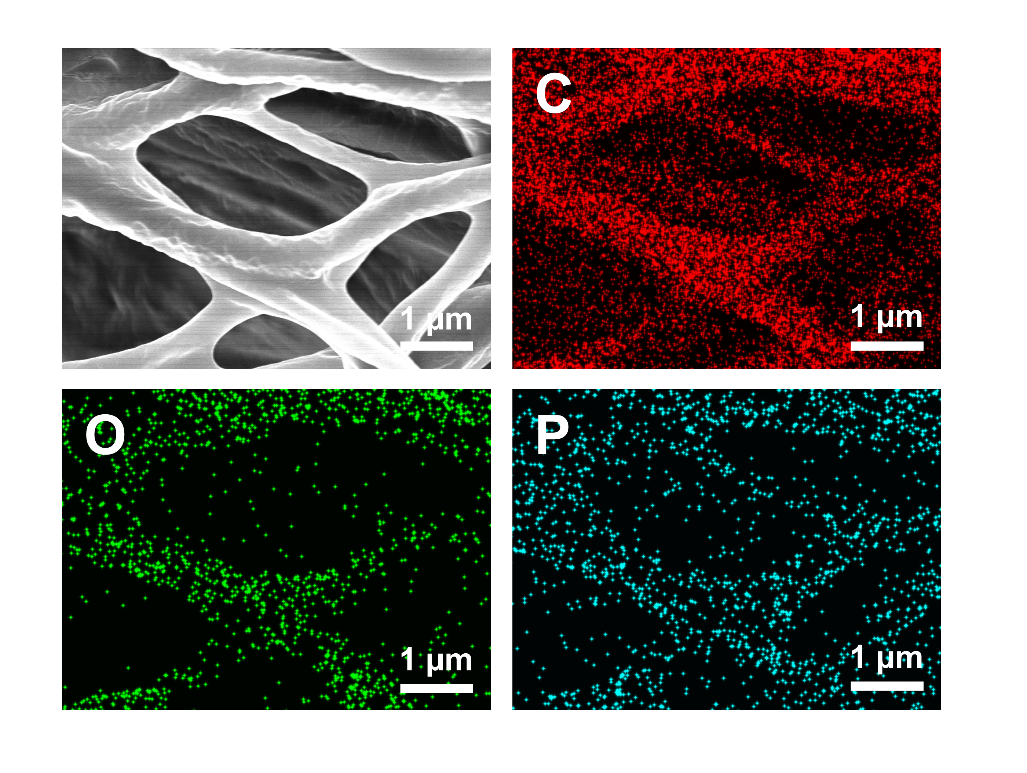


**Figure S10.** SEM image and elemental mapping of JPLCL/ZnO patch.


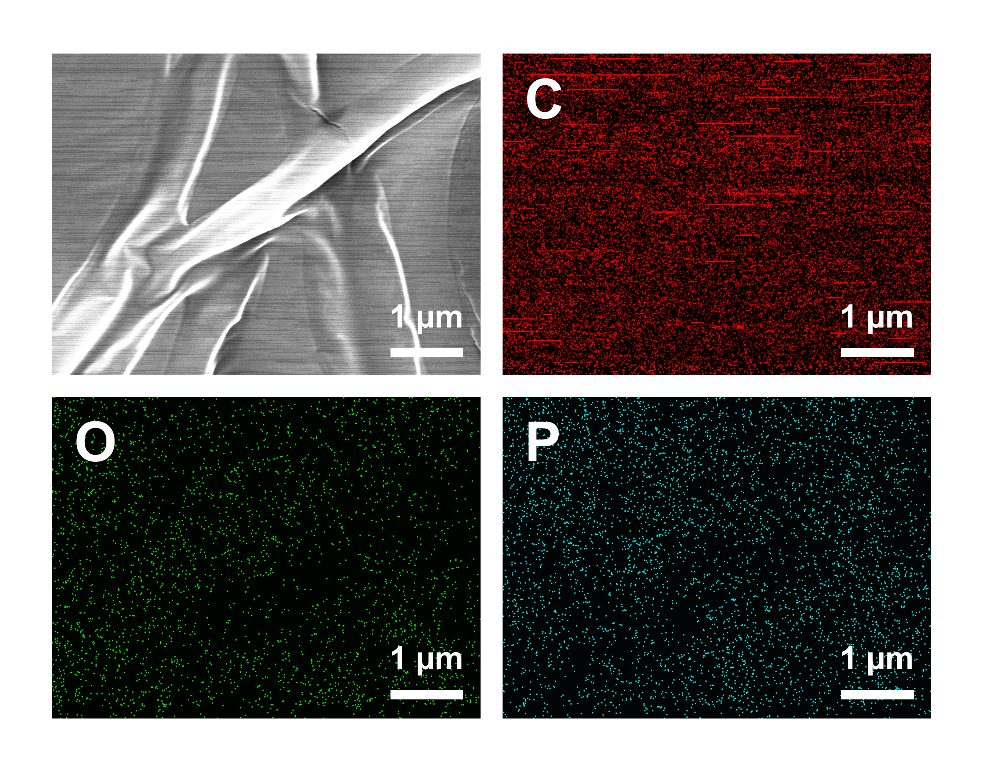


**Figure S11.** SEM image and elemental mapping of BPLCL/ZnO patch.





**Figure S12.** Output voltages of JPLCL/ZnO patches with different ZnO contents.


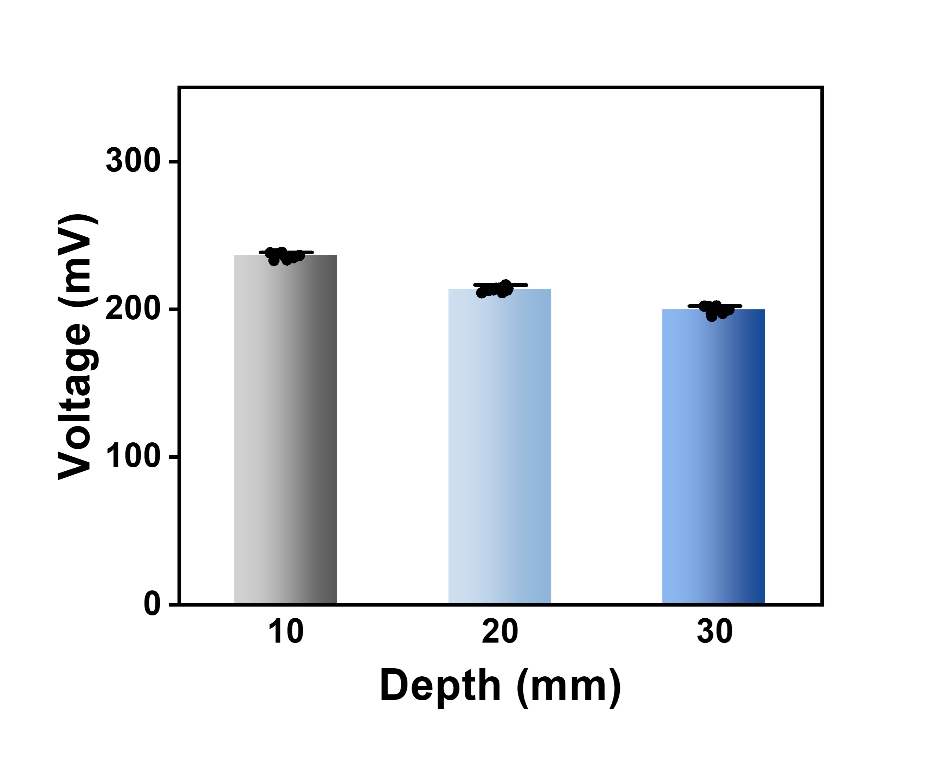


**Figure S13.** Output voltages of JPLCL/ZnO patch implanted at different depths of porcine tissue.


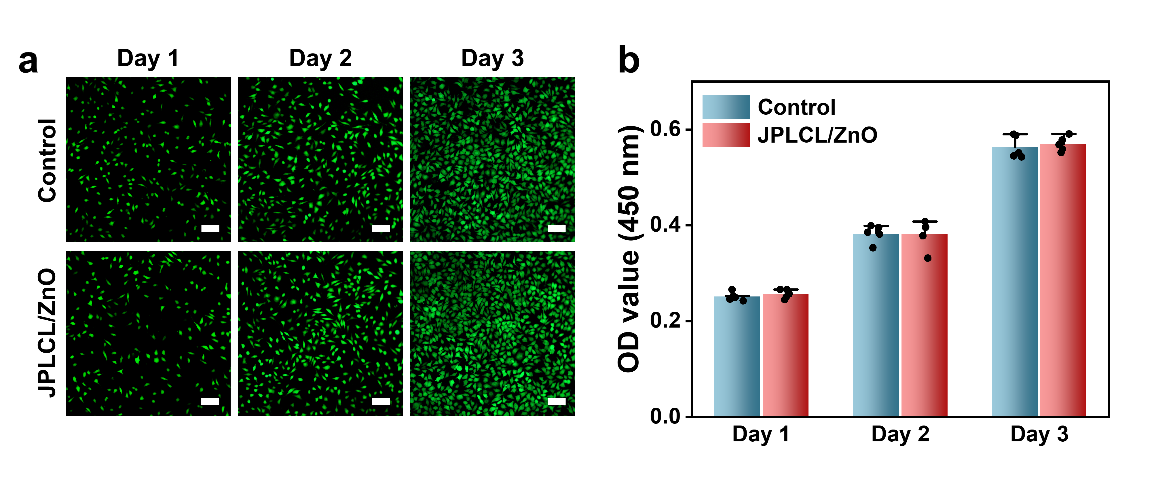


**Figure S14.** In vitro fluorescence images (a) and CCK-8 assay (b) of L929 fibroblasts cultured after 1, 2, and 3 days in DMEM media (control) and JPLCL/ZnO extract (scale bars: 100 µm).


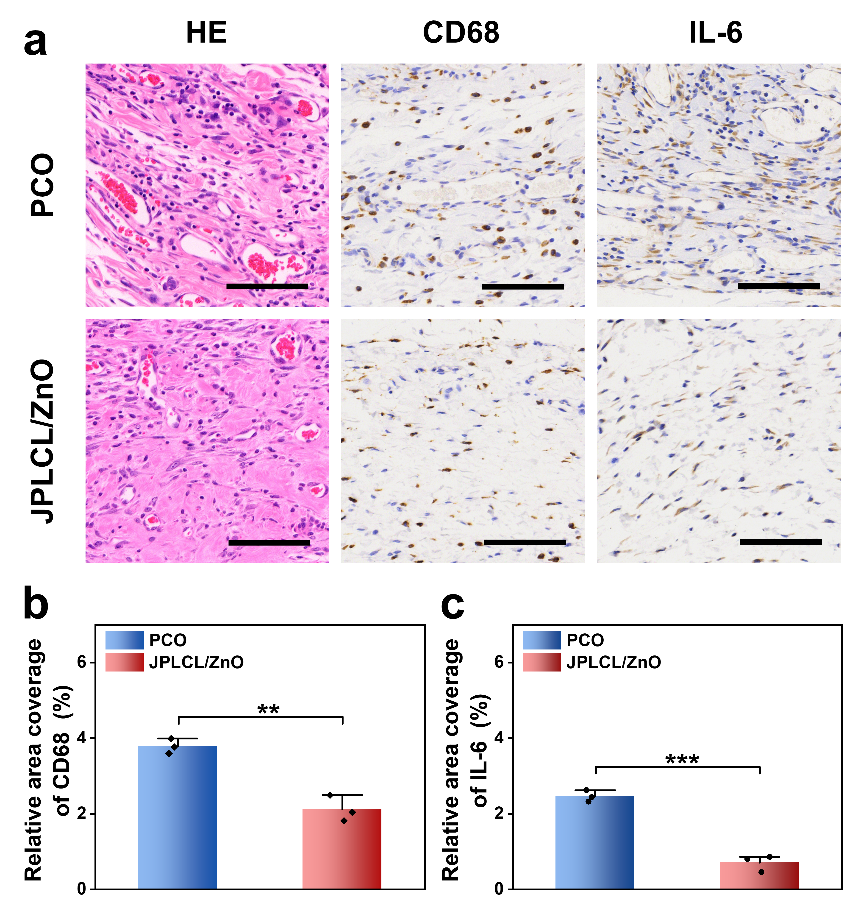


**Figure S15.** a) Images of HE staining and immunohistochemical staining of CD68 and IL-6 for the PCO and JPLCL/ZnO groups after rat subcutaneous implantation for 5 days (scale bars: 100 µm). b, c) Quantitative analysis of CD68 (b) and IL-6 (c) (*n* = 3 independent samples; Student’s *t* test; two-tailed *P* = 0.002 for CD68 and two-tailed *P* < 0.001 for IL-6; error bars = SD; data are presented as mean ± SD).

**
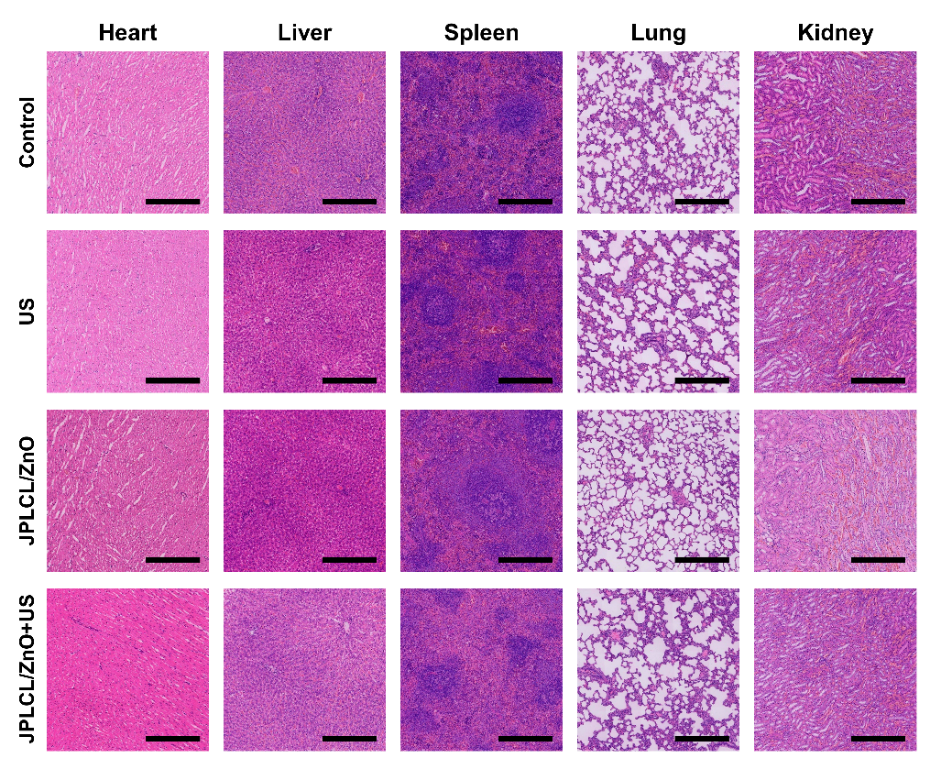
**

**Figure S16.** Images of HE staining of major organs for the control, US, JPLCL/ZnO, and JPLCL/ZnO+US groups (scale bars: 400 µm).


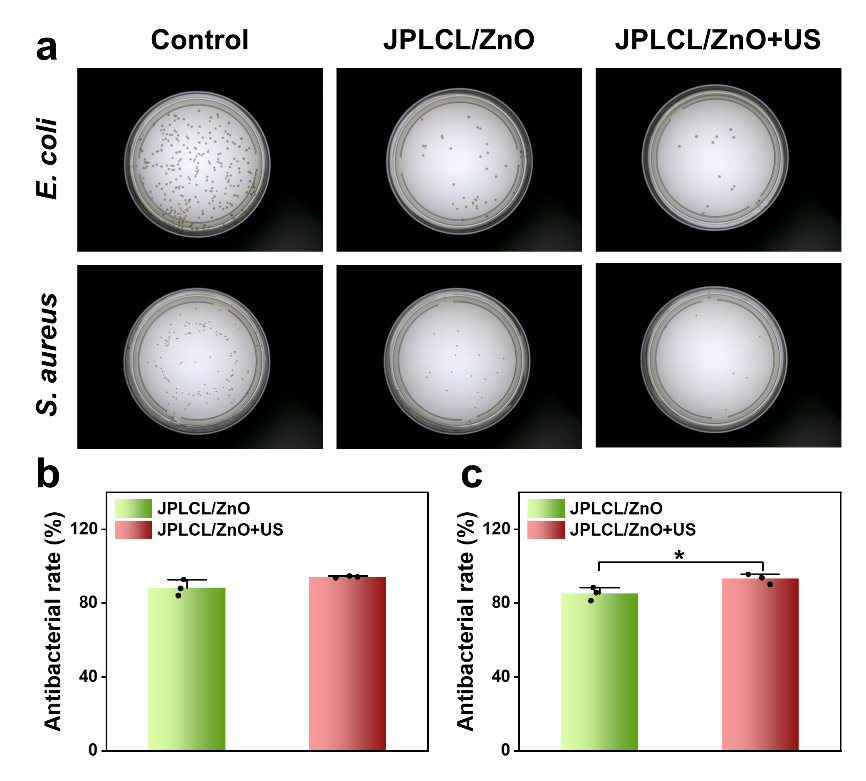


**Figure S17.** a) Images of bacterial colonies formed by *E. coli* and *S. aureus*. b, c) The antibacterial rate of the JPLCL/ZnO and JPLCL/ZnO+US groups for *E. coli* (b) and *S. aureus* (c) (*n* = 3 independent samples; Student’s *t* test; two-tailed *P* = 0.037; error bars = SD; data are presented as mean ± SD).


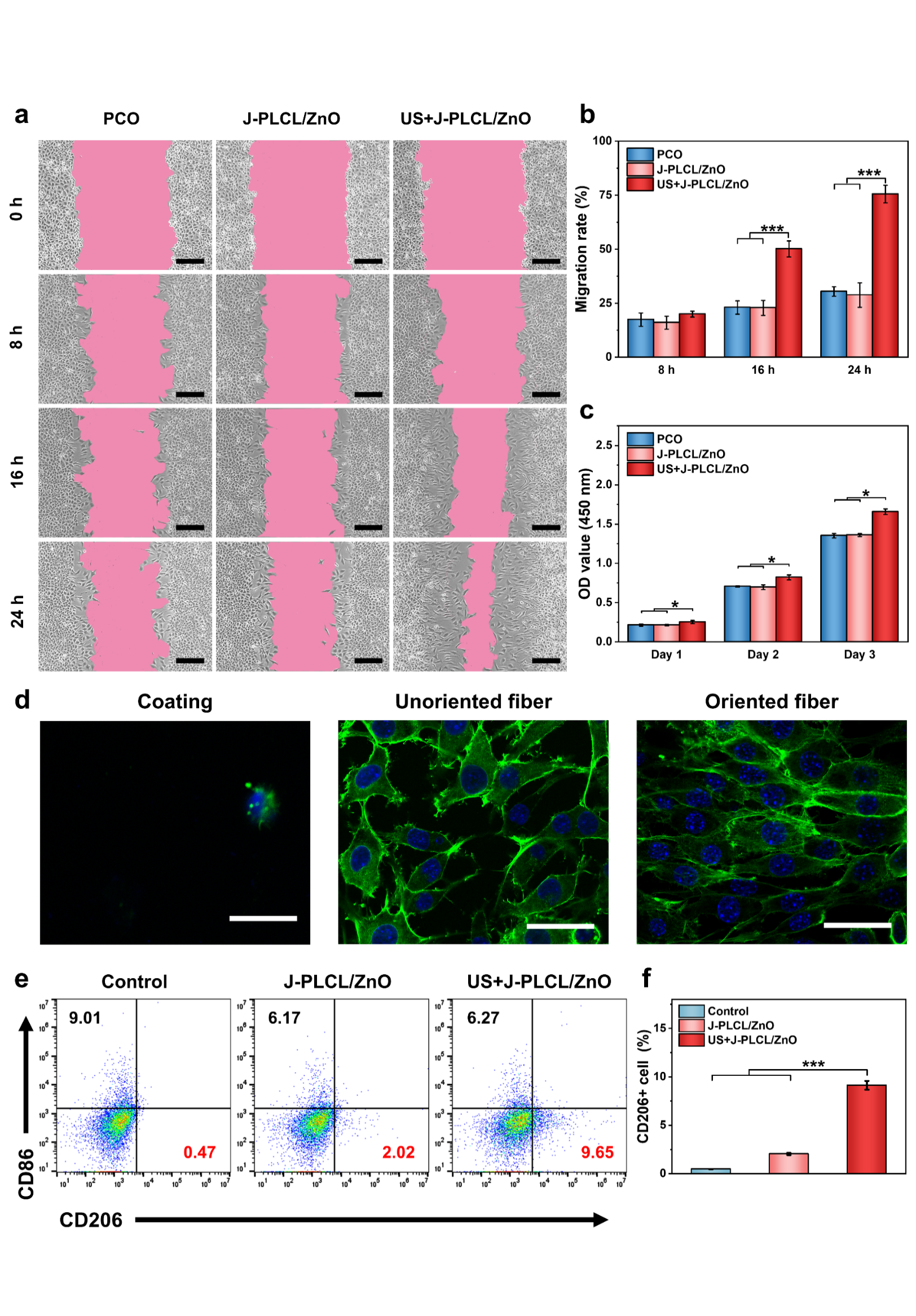


**Figure S18.** Fluorescence image of L929 fibroblasts cultured on random JPLCL/ZnO patch for 1 day (scale bar: 40 µm).


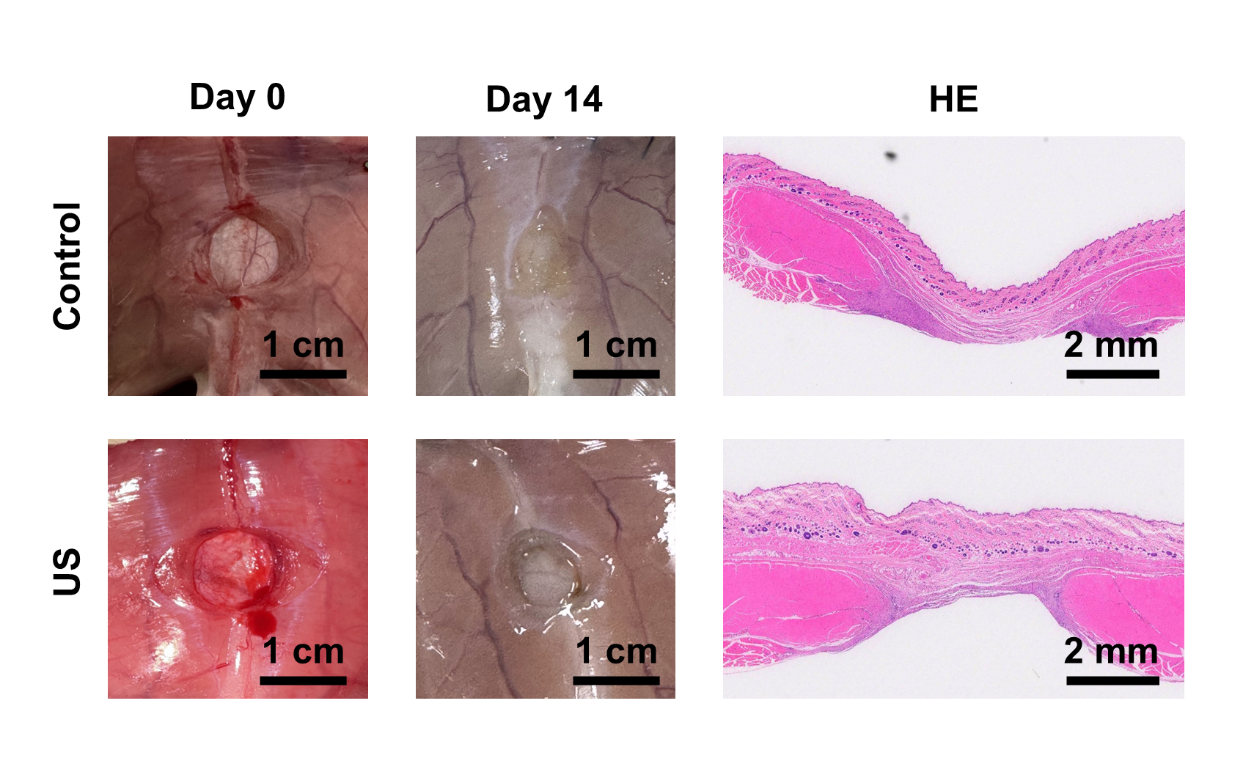


**Figure S19.** Digital photos and HE staining images of abdominal wall defect in the control and US groups.

**
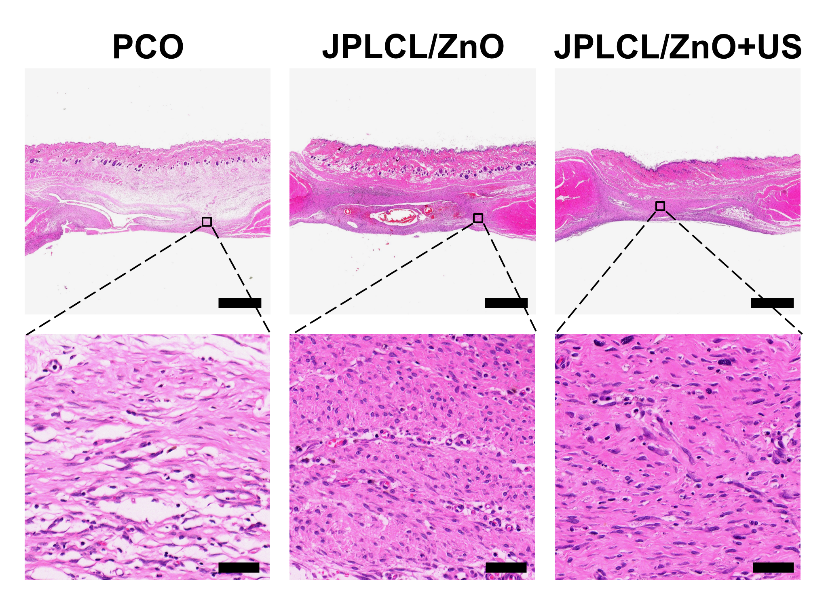
**

**Figure S20.** Images of HE staining for the PCO, JPLCL/ZnO, and JPLCL/ZnO+US groups in a rat abdominal wall defect model (scale bars: 2 mm in row 1, 50 µm in row 2).


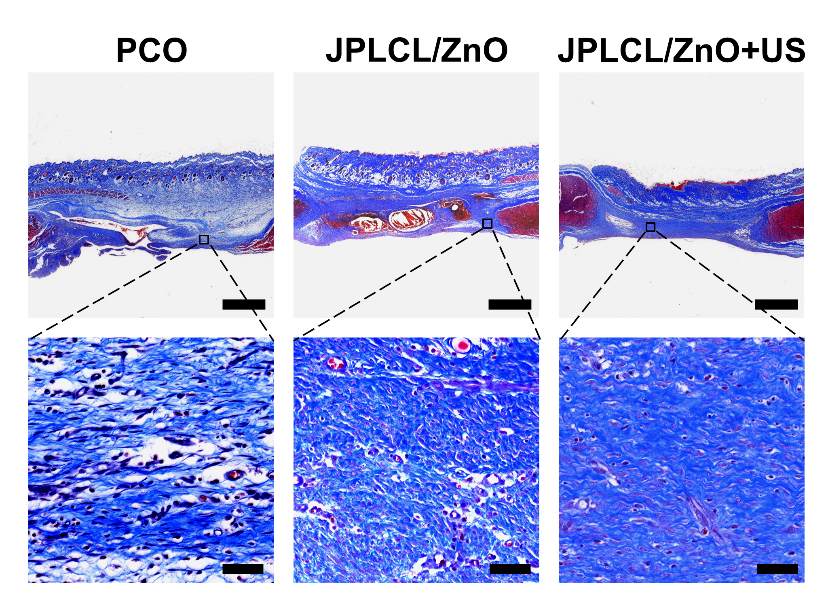


**Figure S21.** Images of Masson staining for the PCO, JPLCL/ZnO, and JPLCL/ZnO+US groups in a rat abdominal wall defect model (scale bars: 2 mm in row 1, 50 µm in row 2).


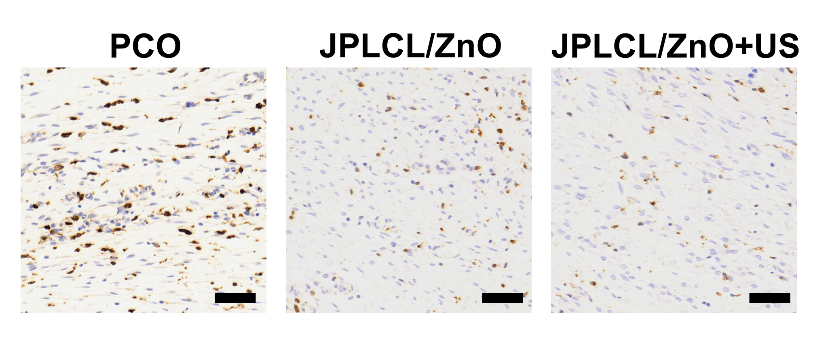


**Figure S22.** Images of immunohistochemical staining of CD68 for the PCO, JPLCL/ZnO, and JPLCL/ZnO+US groups in a rat abdominal wall defect model (scale bars: 50 µm).


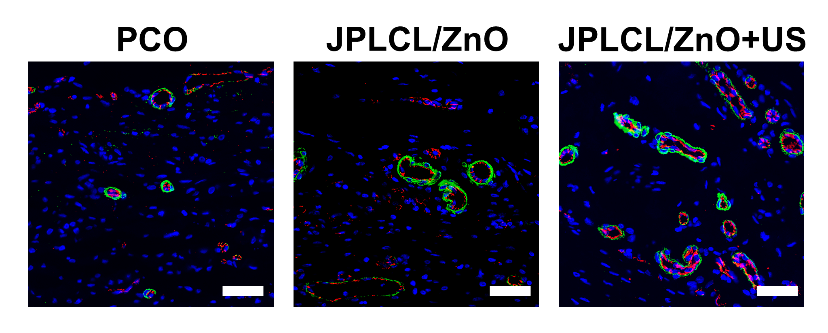


**Figure S23.** Images of immunofluorescence of CD31/α-SMA for the PCO, JPLCL/ZnO, and JPLCL/ZnO+US groups in a rat abdominal wall defect model (scale bars: 50 µm).

**Table S1.** Sample compositions of JPLCL/ZnO patches with different orientation stacking ratios. *Note: 0° and 90° refer to the fiber collection directions during electrospinning, and the listed values represent the preset input volumes (mL) used in sample preparation.*

| **Sample** | **Volume**  **(0°, mL)** | **Volume**  **(90°, mL)** | **Volume ratio**  **(0°:90°)** |
| --- | --- | --- | --- |
| JPLCL/ZnO | 80.00 | 0 | / |
| JPLCL/ZnO (10:1) | 72.73 | 7.27 | 10:1 |
| JPLCL/ZnO (5:1) | 66.67 | 13.34 | 5:1 |
| JPLCL/ZnO (3:1) | 60.00 | 20.00 | 3:1 |
| JPLCL/ZnO (1:1) | 40.00 | 40.00 | 1:1 |
